# The RNA Binding Protein Bcas2 is Required for Antibody Class Switch in Activated‐B Cells

**DOI:** 10.1002/EXP.70015

**Published:** 2025-02-16

**Authors:** Yu Chen, Siyuan Sun, Chenxu Lu, Yixuan Li, Bing Fang, Xiangfeng Tang, Xuepeng Li, Weiru Yu, Yumei Lei, Longjie Sun, Ming Zhang, Jiazeng Sun, Ping Liu, Yongting Luo, Xingwang Zhao, Jing Zhan, Libing Liu, Rong Liu, Jiaqiang Huang, Ziwei Yi, Yifei Yu, Weihan Xiao, Zheng Ding, Lei Li, Dan Su, Fazheng Ren, Changchang Cao, Ran Wang, Wenbiao Shi, Juan Chen

**Affiliations:** ^1^ Key Laboratory of Precision Nutrition and Food Quality Department of Nutrition and Health China Agricultural University Beijing China; ^2^ National Engineering Laboratory for Birth Defects Prevention and Control of Key Technology Beijing Key Laboratory of Pediatric Organ Failure Department of Pediatrics The Seventh Medical Center of PLA General Hospital Beijing China; ^3^ College of Food Science and Engineering Bohai University Liaoning China; ^4^ State Key Laboratory of Animal Biotech Breeding College of Biological Sciences China Agricultural University Beijing China; ^5^ School of Food and Health Beijing Technology and Business University Beijing China; ^6^ State Key Laboratory of Stem Cell and Reproductive Biology Institute of Zoology Chinese Academy of Sciences Beijing China; ^7^ Department of Chemistry and Chemical Biology Cornell University Ithaca New York USA; ^8^ State Key Laboratory of Cardiovascular Disease Fuwai Hospital National Center for Cardiovascular Diseases Chinese Academy of Medical Sciences and Peking Union Medical College Beijing China

**Keywords:** alternative splicing, Bcas2, class switch recombination, CLIP‐seq, hyper‐IgM syndrome type 1, RNA binding proteins

## Abstract

In children, hyper‐IgM syndrome type 1 (HIGM1) is a type of severe antibody disorder, the pathogenesis of which remains unclear. The antibody diversity is partially determined by the alternative splicing (AS) in the germline, which is mainly regulated by RNA‐binding proteins, including Breast cancer amplified sequence 2 (Bcas2). However, the effect of Bcas2 on AS and antibody production in activated B cells, the main immune cell type in the germline, remains unknown. To fill this gap, we created a conditional knockout (cKO, B cell‐specific AID‐Cre *Bcas2* ^fl/fl^) mouse model and performed integrated mechanistic analysis on alternative splicing (AS) and CSR in B cells through the RNA‐sequencing approach, cross‐linking immunoprecipitation and sequencing (CLIP‐seq) analysis, and interactome proteomics. The results demonstrate that *Bcas2‐*cKO significantly decreased CSR in activated B cells without inhibiting the B cell development. Mechanistically, Bcas2 interacts with SRSF7 at a conservative circular domain, forming a complex to regulate the AS of genes involved in the post‐switch transcription, thereby causing broad‐spectrum changes in antibody production. Importantly, we identified GAAGAA as the binding motif of Bcas2 to RNAs and revealed its essential role in the regulation of Bcas2‐dependent AS and CSR. In addition, we detected a mutation of at the 3’UTR of *Bcas2* gene in children with HIGM1 and observed similar patterns of AS events and CSR in the patient that were discovered in the *Bcas2‐*cKO B cells. Combined, our study elucidates the mechanism by which Bcas2‐mediated AS affects CSR, offering potential insights into the clinical implications of Bcas2 in HIGM1.

## Graphical abstract

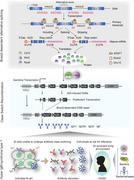

## 1 Introduction

B cells are the major type of immunocytes essential to the adaptive immunity and the development of autoimmune diseases. Once activated by antigens, B cells become mature and express immunoglobulin M (IgM)‐ and IgD‐isotype B cell antigen receptors before they enter the germinal center (GC) [1]. Mature B cells produce a highly variable repertoire of antibodies, including IgE, IgA and IgG, that are designed to neutralize a vast array of pathogens. The key mechanism determining the diversity of antibodies is the antibody class switch recombination (CSR), which involves DNA rearrangement within the Ig heavy chain (IgH) loci located on chromosomes [2]. An IgH locus encompasses a set of heavy chain constant (C_H_) region exons, spanning approximately 220 kilobase (kb) of genomic sequence. These exons determine the isotype and effector function of antibodies. Default switching of the constant (*C_μ_*) region to downstream C_H_ region exons (*C_γ_*, *C_ε_*, or *C_α_*) occurs at the DNA level [3]. The antibody CSR usually occurs on a specific intronic region in B cell germlines termed as the “switch (S) region”. Several factors, such as activation‐induced cytidine deaminase (AID), uracil‐DNA glycosylase (UNG), and apurinic/apyrimidinic endonuclease 1 (APE1), can target the S region and facilitate DNA breaks, contributing to the recombination of the exons which encode variable (V) regions and C_H_ regions of Ig [2, 4–6].

Several cytokines and exogenous stimuli, such as interleukin‐4 (IL‐4), CD40/CD40L, and lipopolysaccharide (LPS), can induce CSR and the generation of germline transcripts (GLTs) in B cells [7–9, 10]. GLTs are categorized as long non‐coding RNAs containing the S region [11, 12, 13]. During CSR, GLTs are induced to be generated in concomitant with DNA rearrangement of IgH genes, for which targeting GLTs has been acknowledged as an approach to modulating CSR [13, 14, 15]. CSR is a highly regulated process that involves multiple RNA‐binding proteins (RBPs), which modulate the splicing, stability, and translation of mRNAs critical for the CSR pathway. For example, the RNA helicase DEAD‐Box Helicase 1 (DDX1) binds to the S region RNA/DNA G‐quadruplexes, assisting to form R‐loop structure and promoting CSR in B cells [16]. Regulator of differentiation 1 (ROD1) plays a crucial role in directing AID to specific genomic loci, thereby facilitating DNA rearrangement during immune responses [17]. Another study identified one splicing factor Phf5a/Sf3b14b, which can modulate the DNA repair step of CSR [18]. However, the effect of RBPs on regulating the CSR‐related genes through alternative splicing (AS) has not been sufficiently studied yet. Previous studies have reported the regulation of GLTs through AS [19] in the developing GC B cells [20, 21, 22]. AS results in exclusion, inclusion or modification of exons, thereby regulating the gene expression and final protein diversity. The AS events have been observed in different types of B cells [17, 20–22], but evidence of AS function in activated B cells is lacking. Due to the indispensable role RBPs play in the process of AS [23, 24, 25, 26, 27], it is of great significance to investigate the regulation of RBP on AS in CSR of the activated B cells.

Breast cancer amplified sequence 2 (Bcas2), also known as splicing factor 27 (SPF27) [28], is markedly upregulated on the gene expression level in human breast cancer cells [29]. Bcas2 per se is an RBP and can serve as a highly conserved component of the CDC5L/Prp19 splicing complex, which is involved in the assembly of the mRNA splicing body [28, 30, 31]. Several studies have demonstrated the regulation of Bcas2 on AS in sperm cells [32, 33], oocytes [34], pancreatic β cells [35] and several types of cancer cells [36, 37]. Recently, it has been shown that Bcas2 is vital for the maintenance of hematopoietic stemness through the regulation of AS on p53‐targeted genes in zebrafish [38]. These studies underline the impact of Bcas2 on regulating AS in cell proliferation and cell secretion. To achieve the production of diversified antibodies, AS could be precisely executed by one or a group of RBPs. Therefore, it is crucial to explore the impact of Bcas2 on the antibody CSR in B cells and uncover the underlying mechanisms.

Here, we performed integrated mechanistic studies on the effect of Bcas2 on the AS and CSR in B cells. We found that specific deletion of Bcas2 in activated B cells contributes to significant changes in the expression of a series of genes, which are related to B cell activation and DNA recombination, as well as significant alterations in AS events of genes associated with RNA transcription and splicing. These AS changes were achieved through Bcas2‐specific binding motifs. Based on the protein interactome analysis of Bcas2, we identified a couple of interactive proteins of Bcas2, SRSF7, and DHX15, which bind Bcas2 to regulate AS and CSR. In addition, we analyzed RNA collected from patients with hyper‐IgM syndrome type 1 (HIGM1); a similar pattern of CSR and AS alterations was found in both patient samples and the *Bcas2*‐cKO mice model. We thereby established the connection between the AS regulation of Bcas2 and the etiology of HIGM1, and laid a theoretical foundation of HIGM1.

## 2 Results

### 2.1 Ablation of *Bcas2* Impairs the Antibody CSR in Murine B Cells

**FIGURE 1:**
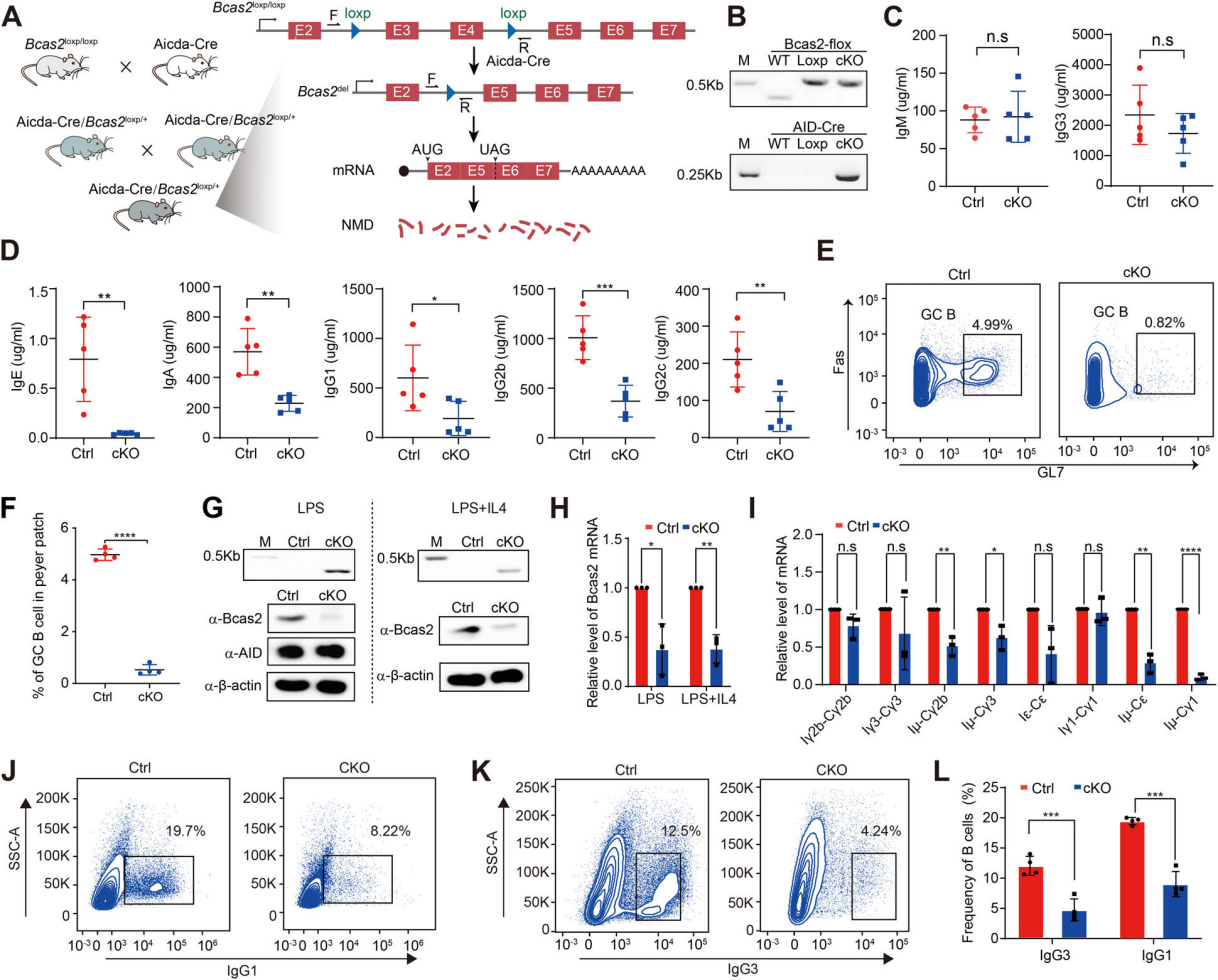
AID‐Cre knockdown of Bcas2 reduces the ability of B cells to produce antibodies. (A) Diagram of conditional Bcas2 (targeting allele, obtained through gene editing Bcas2 WT allele using targeting vector), and Cre‐deleted Bcas2 allele (Bcas2 KO allele). PCR primers are indicated. (B) Genotype results of Bcas2^fl/fl^ AID Cre/+ (*Bcas2*‐cKO) mice. (C) Concentrations of IgM and IgG3 in 7‐week‐old WT and *Bcas2*‐cKO mice serum. Each symbol represents an individual mouse and horizontal lines indicate the mean. (D) Concentrations of IgA, IgE, IgG1, IgG2b and IgG2c in 7‐week‐old WT and *Bcas2*‐cKO mice serum. Each symbol represents individual mice and horizontal lines indicate the mean. (E) Flow cytometric analysis for the proportion of GC B cells in Peyer's patches of WT and *Bcas2*‐cKO mice. (F) Quantification of GC B shown in (D). Each symbol represents B cell cultures from individual mice (*n* ≥ 4, mean ± SD). (G) Genomic DNA PCR analysis and western blot of LPS and LPS+IL4 stimulated WT and Bcas2^fl/fl^ AID Cre/+ (*Bcas2*‐cKO) mice (day 3). (H) Quantitative PCR analysis of WT and *Bcas2*‐cKO B cell mRNA after 72 h of LPS and LPS+IL4 stimulation. Bcas2 expression levels were normalized to β‐actin transcripts in Ctrl of LPS and LPS+IL4 stimulation (*n* = 3, mean ± SD). (I) Quantitative PCR analysis of mRNA after 72 h in LPS and LPS + IL4 stimulation. Expression levels of variable and constant regions of different Ig were normalized to β‐actin transcripts and Ctrl of LPS and LPS+IL4 stimulation (*n* = 3, mean ± SD). (J) Flow cytometric analysis for surface IgG1 expression after LPS+IL4 stimulation. (K) Flow cytometric analysis for surface IgG3 expression after LPS stimulation. (L) Quantification of IgG1 and IgG3 for LPS and LPS+IL4 cultures shown in (J,K) (*n* ≥ 4, mean ± SD).

To decipher the function and mechanisms of Bcas2 in antibody diversification, we first examined the expression of Bcas2 in different types of B cells. Bcas2 was ubiquitously expressed in the immune tissues including the spleen, bone marrow, thymus, liver, Peyer's patches, lymph nodes, and lungs, irrespective of sex and age (Figure S1A–C). Bcas2 was present at significant levels in B cells at different stages, including follicular B cells (Fo B) which are essential for antibody production (Figure S1D). B cells are the exclusive site for antibody production, in which a vast variety of types of immunoglobulins (Ig) can be generated in response to different antigens. Therefore, we first examined the effects of B cell‐specific *Bcas2* deletion on the antibody CSR in vivo. By crossing the mice harboring loxP‐flanked *Bcas2* alleles (*Bcas2* ^fl/fl^) [39] with the AID‐Cre mice, we generated a conditional knockout (cKO, AID‐Cre *Bcas2* ^fl/fl^) mouse model in which deletion of the *Bcas2* gene was initiated at the activated B cells (Figure 1A). The ablation of exons 3 and 4 will introduce a stop codon in exon 6 and trigger the mRNA degradation by nonsense‐mediated RNA decay. The genotyping analysis confirmed the successful insertion of the loxP sites at the intronic regions of *Bcas2* and the expression of AID‐Cre in *Bcas2*‐cKO mice (Figure 1B). Next, we found that the levels of high‐affinity antibodies, including IgA, IgE, IgG1, IgG2b and IgG2c, were significantly decreased in the serum of the *Bcas2*‐cKO mice compared to their control littermates (*Bcas2* ^fl/fl^), while the levels of IgM and IgG3 did not alter upon conditional knockout of *Bcas2* in B cells (Figure 1C,D). Consistent with the changes in circulating Ig, the proportion of GC B cells and IgA^+^ GC B cells in the Peyer's patches of the *Bcas2*‐cKO mice was significantly lower than that in the control group (Figure 1E,F). Moreover, the percentage of IgA^+^ GC B cells of the *Bcas2*‐cKO mice was also significantly reduced (Figure S2D). These data suggest that *Bcas2* is essential in the production of high‐affinity, class‐switched antibodies.

Since CSR is an indispensable process of antibody production in B cells, we further determined whether Bcas2 affects the antibody CSR ex vivo. We isolated primary B cells from the spleen of the *Bcas2*‐cKO and control mice, stimulating these cells with LPS or LPS+IL‐4 to induce class switching of different antibodies ex vivo. Upon the stimulation of LPS and LPS+IL4, the exons of the *Bcas2* gene were excised from the genome in the primary B cells of the *Bcas2*‐cKO mice (Figure 1G), resulting in significant decreases in both mRNA and protein levels of Bcas2 (Figure 1G,H), whereas the AID expression was not changed (Figure 1G). In response to LPS or LPS plus IL‐4 stimulation, all post‐switch transcripts were significantly reduced in *Bcas2*‐cKO B cells while germline transcripts did not exhibit any significant change (Figure 1I), suggesting a B cell‐specific CSR defect in the absence of Bcas2. Consistent with this finding, *Bcas2* deletion in activated B cells markedly reduced populations of IgG1^+^ and IgG3^+^ B cells compared to the control group (Figure 1J–L). By contrast, *Bcas2*‐deficient B cells displayed significant decreases in populations of IgE^+^ and IgG2b^+^ B cells (Figure S2A–C).

We also employed the CH12 cell line, which has been demonstrated to undergo CSR for the efficient production of IgA upon cytokine stimulation [40]. We generated Bcas2‐depleted CH12 cells by transient knockdown with siRNA (Figure S1E–G). The population of IgM^−^ IgA^+^ positive cells dropped dramatically in the *Bcas2*‐depleted cells in response to CD40, IL‐4, and TGF‐β stimulus, as compared to the control group (Figure S1H,I). In line with this, the levels of IgA in the culture media supernatant were significantly decreased in response to the *Bcas2* knockdown, while the IgM levels were not affected (Figure S1J). Besides, *Bcas2* depletion led to a significant reduction of IgA post‐switch transcription upon the stimulation, whereas the level of germline transcription remained unchanged (Figure S1K). A rescue experiment was conducted for *Bcas2*, revealing that protein expression was successfully restored. Additionally, the population of IgM^−^ IgA⁺ cells was significantly elevated compared to the *Bcas2* knockdown group, showcasing its impact on antibody production (Figure S1L,M). To further investigate the effects of Bcas2 on the antibody CSR in response to different antigens in vivo, we immunized the *Bcas2*‐cKO and control mice with ovalbumin (OVA) and keyhole limpet hemocyanin (NP‐KLH). Then we analyzed the serum antibody profile with the immunization of OVA. After the immunization with OVA, the levels of antigen‐specific Ig including IgA, IgE and multiple types of IgGs, but not IgM, were significantly lower in the serum of the *Bcas2*‐cKO mice, as compared to the control group (Figure S2E). In contrast, the *Bcas2*‐cKO mice immunized with NP‐KLH showed no changes in levels of IgM, IgG3, and IgA, whilst levels of IgG subtypes and IgE were reduced (Figure S2E). Collectively, these results reveal that Bcas2 may have broad‐spectrum regulatory effects on the antibody CSR in B cells.

### 2.2 Deficient of *Bcas2* Does not Affect B Cell Development and Proliferation

Since CSR has been shown its association with cell division [41], we determined the effects of *Bcas2* deletion on the proliferation and development of B cells. The *Bcas2*‐cKO adult mice displayed similar body weight growth compared with their control littermates, regardless of their sex (Figure S3A). Similar populations of B cells at different stages of differentiation in the spleen and bone marrow were observed between the *Bcas2*‐cKO and control (Figure S3B–E), suggesting that deletion of *Bcas2* did not affect the B cell development. To assess whether Bcas2 knockout affects the proliferation of activated B cells, we utilized CFSE to label B cells and subsequently stimulated these CFSE‐labeled B cells with LPS and IL‐4. The results indicate that Bcas2 knockout did not impact the proliferation of activated B cells, but the proportion of IgG1 in proliferating B cells was significantly reduced following Bcas2 knockout (Figure S3F). A similar reduction in IgG1 CSR levels was observed in cells undergoing different numbers of cell divisions, further proving the above results (Figure S3G). Of note, the reduced serum antibody levels were not due to defects in cell proliferation. Overall, our data show that ablation of *Bcas2* in the activated B cells has a very limited influence on the B cell development.

### 2.3 Transcription Profiling in Bcas2‐Depleted B Cells

**FIGURE 2:**
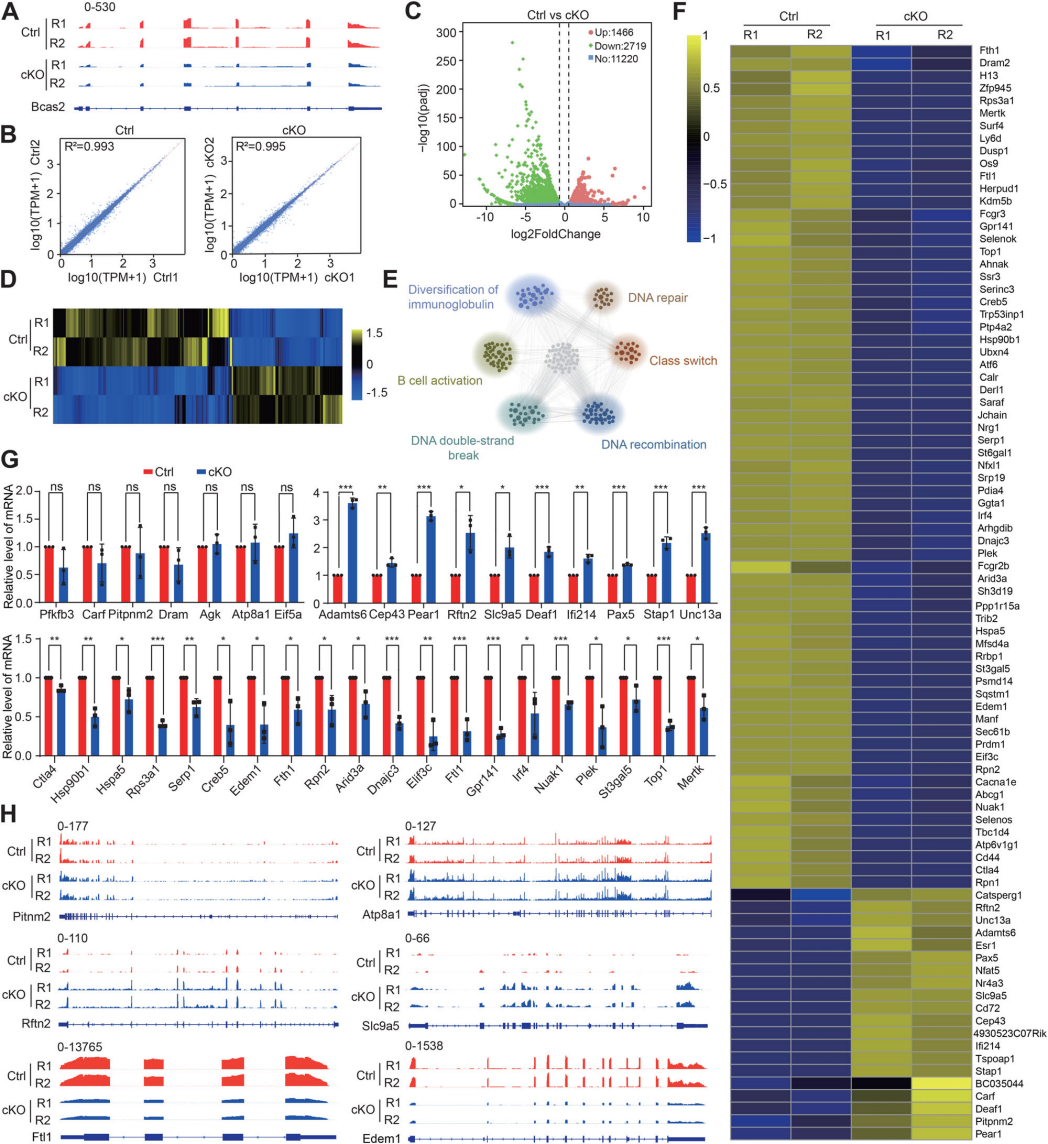
Bcas2 regulates mRNA abundance in activated‐B cells. (A) The expression of Bcas2 is shown as coverage tracks in LPS+IL4 stimulated WT and *Bcas2*‐cKO B cells. (B) Pearson correlation analysis shows the coefficients between two replicates of WT and *Bcas2*‐cKO mice in the RNA‐seq data. (C) Volcano map displaying the distribution of differentially expressed genes from RNA‐seq data. The abscissa in the figure represents the gene fold change in LPS+IL4 stimulated WT and *Bcas2*‐cKO B cells. |FoldChange| ≥ 1.5. Padj ≤ 0.05. The ordinate indicates the significance of gene expression differences between LPS+IL4 stimulated WT and *Bcas2*‐cKO B cells. Upregulated genes are shown as red dots, and downregulated genes are shown as green dots. (D) Cluster heatmap of differentially expressed genes. The abscissa is the genotype, and the ordinate is the normalized FPKM (fragments per kilobase million) value of the differentially expressed gene. Yellow indicates a higher expression level, while blue indicates a lower expression level. (E) Network showing GO enrichment analyses of differentially expressed genes. (F) Heatmap of CSR‐associated, DNA recombination and repair‐associated, and Bcas2‐binding gene expression. (G) The expression of CSR‐associated, DNA recombination and repair‐associated, and Bcas2‐binding genes in LPS+IL4 stimulated WT and *Bcas2*‐cKO B cells. The RT‐qPCR data were normalized to gapdh. The value in the WT group was set as 1.0, and the relative value in the *Bcas2*‐cKO group is indicated (*n* = 3). Unpaired Student's *t* test determined significance and exact *p* value. The points and error bars represent the mean ± SEM. (H) RNA‐seq data represent the expression of CSR‐associated, DNA recombination and repair‐associated, and Bcas2‐binding genes.

To investigate the mechanisms underlying the effects of Bcas2 on CSR in B cells, we performed integrated mechanistic studies ex vivo, including: (1) the RNA‐sequencing (RNA‐seq) approach for whole‐transcriptome analysis and to identify the effect of Bcas2 on AS and gene expression related to CSR; (2) cross‐linking immunoprecipitation and sequencing (CLIP‐seq) analysis to investigate how Bcas2 regulates AS of mRNA; and (3) interactome analysis followed by validation studies to identify the Bcas2‐bound proteins that are essential for AS of target genes engaging in the CSR. We confirmed the decreased expression of *Bcas2* mRNA in LPS+IL‐4‐treated splenic B cells of the *Bcas2*‐cKO mice compared to that in the control group, by visualizing the transcriptome of Bcas2 using the Integrative Genomics Viewer (IGV) [42] (Figure 2A). The whole transcriptome sequencing analysis showed high biological reproducibility within the same groups (Figure 2B and Figure S4A). Expressions for a total of 4185 genes were significantly changed in the *Bcas2*‐cKO group compared with the control group, including 1466 upregulated genes and 2719 downregulated genes (Figure 2C,D and supplementary data Table S4). In contrast to mRNA transcriptome, *Bcas2*‐cKO had limited influence on long non‐coding RNA (lncRNA) transcriptome as 264 out of 26,517 genes had significant changes in the lncRNA expression (158 upregulation and 106 downregulation) (Figure S4B,C).

Gene ontology (GO) enrichment analysis underlines the diversification of Ig, B cell activation and class switch, suggesting that CSR was affected by *Bcas2*‐cKO (Figure 2E), in favor of the previous notion (Figure 1 and Figure S1). Apart from that, *Bcas2*‐cKO had impacts on regulatory activities of DNA double‐strand break, repair and recombination (Figure 2E). The expression of the top 10% upregulated and downregulated genes based on the fold changes in *Bcas2*‐cKO over the control were visualized through heatmap and subsequently validated by qRT‐PCR (unchanged: *Pfkfb3*, *Carf*, *Pitpnm2*, *Dram*, *Agk*, *Atp8a1*, and *Eif5a*; upregulated: *Adamts6*, *Cep43*, *Pear1*, *Rftn2*, *Slc9a5*, *Deaf1*, *Ifi214*, *Pax5*, *Stap1*, and *Unc13a*; downregulated: *Ctla4*, *Hsp90b1*, *Hspa5*, *Rps3a1*, *Serp1*, *Creb5*, *Edem1*, *Fth1*, *Rpn2*, *Arid3a*, *Dnajc3*, *Elif3c*, *Ftl1*, *Gpr141*, *Irf4*, *Nuak1*, *Plek*, *St3gal5*, *Top1*, and *Mertk*) (Figure 2F,G). The IGV results of the gene *Pitnm2*, *Atp8a1*, *Rftn2*, *Slc9a5*, *Ftl1*, and *Edem1* further showed that the expressions of the majority of exons in these genes were markedly altered by *Bcas2* depletion (Figure 2H). The results above indicate that Bcas2 affects CSR in B cells by modulating the splicing of alternative exons for CSR regulation‐associated genes.

### 2.4 Bcas2 Regulates AS in Activated B Cells

**FIGURE 3:**
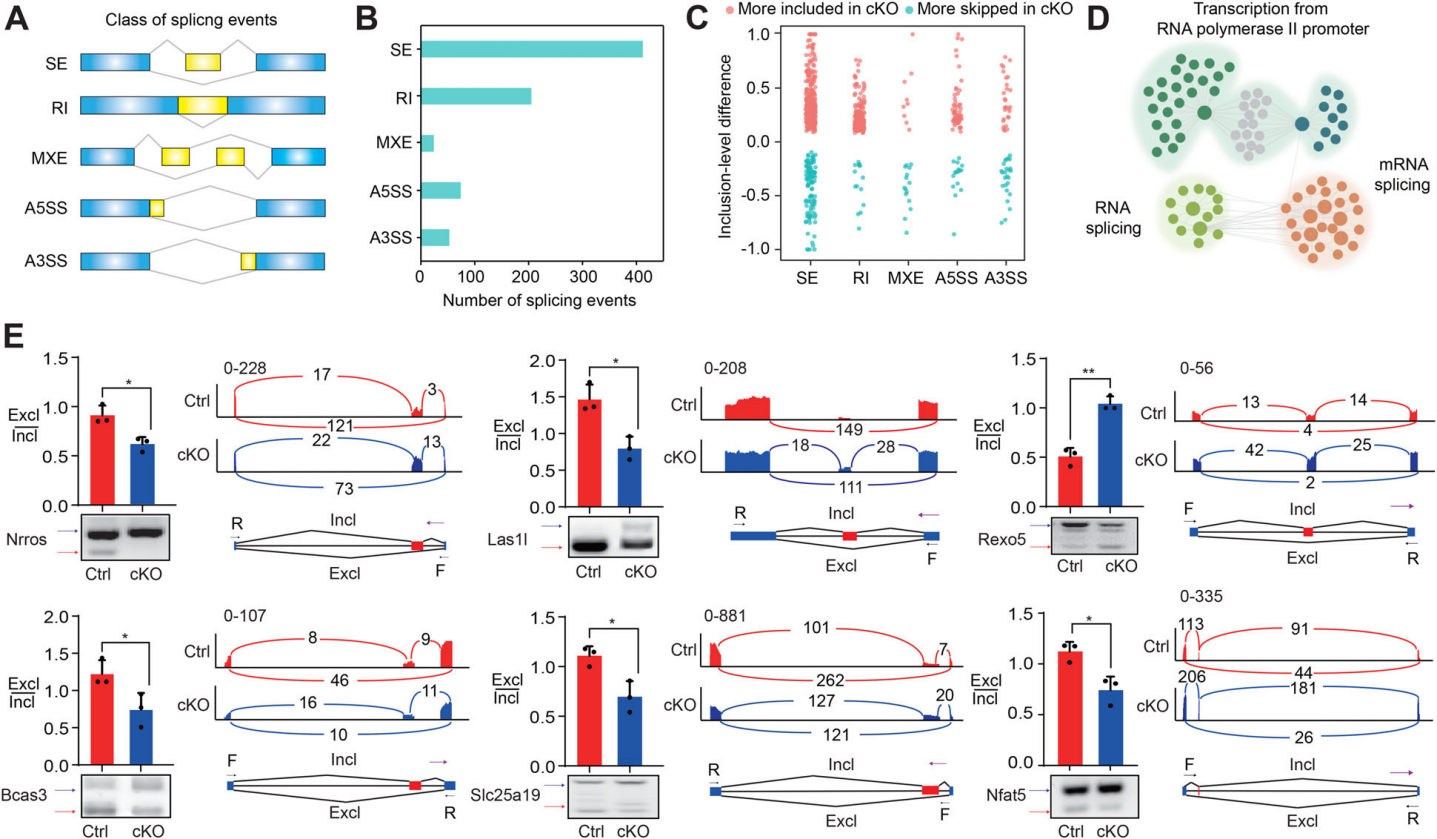
Bcas2 regulates mRNA Alternative splicing. (A) Schematic diagram showing five typical classes of splicing events. (B) Splicing events were analyzed by number. (C) Splicing events were analyzed by exclusion and inclusion. (D) Network showing GO enrichment analyses of aberrant AS genes. (E) Alternative splicing of Msi2/Cd72/‐ in the LPS+IL4 stimulated WT and *Bcas2*‐cKO splenic B cells was analyzed by RT‐PCR (*n* ≥ 3). The ratios of inclusion (Incl) to exclusion (Excl) are shown accordingly. Analysis of CSR‐associated, DNA recombination and repair‐associated, and Bcas2‐binding gene expression and exon–exon junctions.

We next investigate the effect of Bcas2 on AS events in B cells. By employing the Alternative Splicing Detector (ASD) software [43], we found 768 AS events that were significantly altered by *Bcas2*‐cKO in the activated B cells compared to the control group. Among these AS events, 412 events were classified as skipped exons (SEs), 205 events were retained introns (RIs), 24 events were mutually exclusive exons (MXEs), 74 events were 5' splice sites (A5SSs), and 53 events were 3' splice sites (A3SSs) (Figure 3A,B). The *Bcas2*‐cKO B cells exhibited both upregulation and downregulation in all the analyzed types of AS events (Figure 3C). The GO enrichment analysis revealed that gene transcripts that underwent abnormal splicing due to *Bcas2*‐cKO were related to RNA splicing (Figure 3D). Notably, AS analysis suggested abnormal splicing of pre‐mRNAs of *Nrros*, *Las1l*, *Rexo5*, *Bcas3*, *Slc25a19*, and *Nfat5* (Figure 3E), Of which, *Nfat5* has been shown to be associated with CSR and DNA recombination and repair [44]. The *Bcas2* rescue experiment showed alternative splicing events in CH12 cells was recovered in the *Bcas2* rescue group compared to the *Bcas2* knockdown group (Figure S1O). Combined, our data demonstrate that Bcas2 affects AS during the B cell activation.

### 2.5 Bcas2 Binds to RNA Genome‐Widely

**FIGURE 4:**
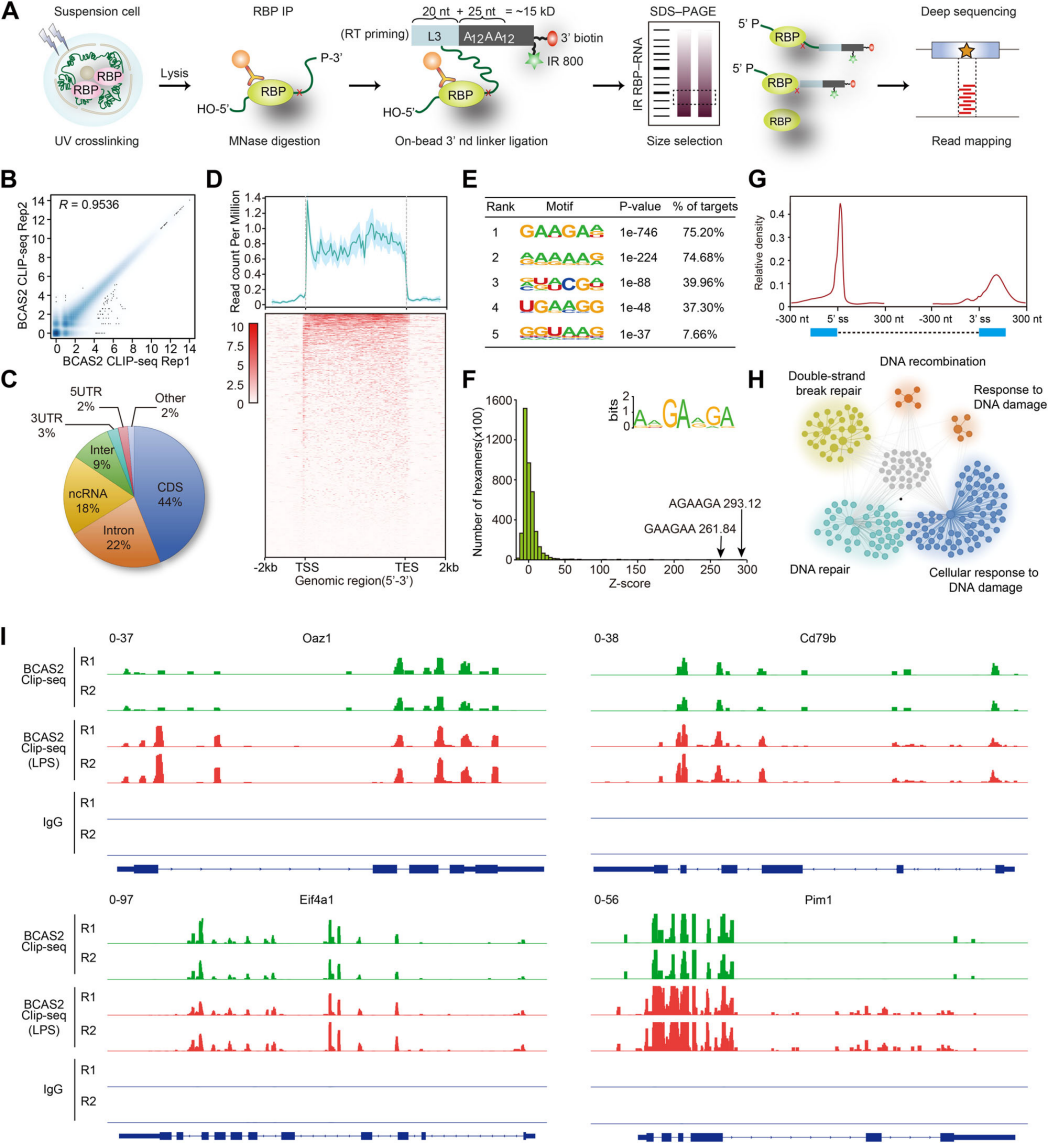
Bcas2 directly binds mRNAs after LPS stimulation. (A) Schematic diagram showing the flow of CLIP‐seq. (B) Pearson's correlation analysis shows the coefficient between two replicates in the Clip‐seq data. (C) Pie chart of sequence regions in which Bcas2 primarily binds. (D) Meta‐analysis showed the binding of Bcas2 in the genomic region between TSS and TES. (E) Enriched Bcas2 binding motifs. The top five enriched motifs are shown. (F) Histogram showing overrepresented Bcas2‐binding motifs identified by CLIP‐seq. The *Z* scores of the top two hexamers are indicated. The insert shows the Bcas2‐binding consensus calculated from the top 20 enriched hexamers. (G) Meta‐analysis showing the relative density of Bcas2 binding to 5'ss and 3'ss. (H) GO enrichment analysis network showing the corresponding genes for Bcas2 binding RNA. (I) The Bcas2‐binding peaks of CSR‐related gene transcripts are shown. Purple arrow, direction of transcription. F, forward primers; R, reverse primers.

Next, we mapped the RNA targets of Bcas2 by CLIP‐seq in B cells in the absence and presence of LPS (Figure 4A). We generated the highly reproducible CLIP‐seq libraries (Figure 4B and Figure S5F). A total of 9990 significant peaks were identified, which were matched by 4300 genes, with 44% and 22% of all detected sequences confined to CDSs and introns of RNA, respectively (supplementary data Table S3). The remaining sequences that were significantly bound to Bcas2 were lncRNA (accounting for 18%), intergenic regions (9%), 3' untranslated regions (3' UTR, 3%) and 5' untranslated regions (5' UTR, 2%) (Figure 4C). Meta‐analysis revealed that Bcas2 primarily binds to 5' UTR at the transcription start site (TSS) as well as 3'UTR at the transcription end site (TES) of genebody in the activated B cells (Figure 4D). The results derived from the Kmer and HOMER software revealed enrichment in GAAGAA‐rich motifs (Figure 4E,F). In addition, Bcas2 primarily bound to the 5' splicing sites and 3' splicing sites of RNA (Figure 4G). The Bcas2‐bound mRNAs were enriched in pathways related to DNA recombination, double‐strand breaks, and DNA damage repair (Figure 4H), indicating that Bcas2 may mediate CSR through direct binding the transcripts of genes involving the CSR process. CLIP‐seq analysis was also conducted on the splenic B cells ex vivo in the absence of LPS treatment. Under such conditions, a total of 3643 significant peaks that correspond to 2119 genes were identified, which were markedly lower than those observed in the LPS treatment. The tendency of Bcas2 binding sites, binding motif as well as gene enrichment in the absence of LPS were comparable to those with LPS stimulation (Figure S5A–E). By contrast, the abundance of multiple exons of Bcas2 bound mRNAs, including *Oaz1*, *Cd79b*, *Elif4a1*, *Pim1*, *Adrbk1*, *Cdh4*, *Ptpn6*, *Cd22*, *RPI26* and *Cxxc1*, was significantly augmented in response to LPS (Figure 4I and Figure S5G). The results above suggest that Bcas2 may affect CSR by directly binding to different regions of RNA genome‐widely with sequence‐specificity.

### 2.6 Bcas2 Regulates AS Genome‐Wide by Binding RNA Directly

**FIGURE 5:**
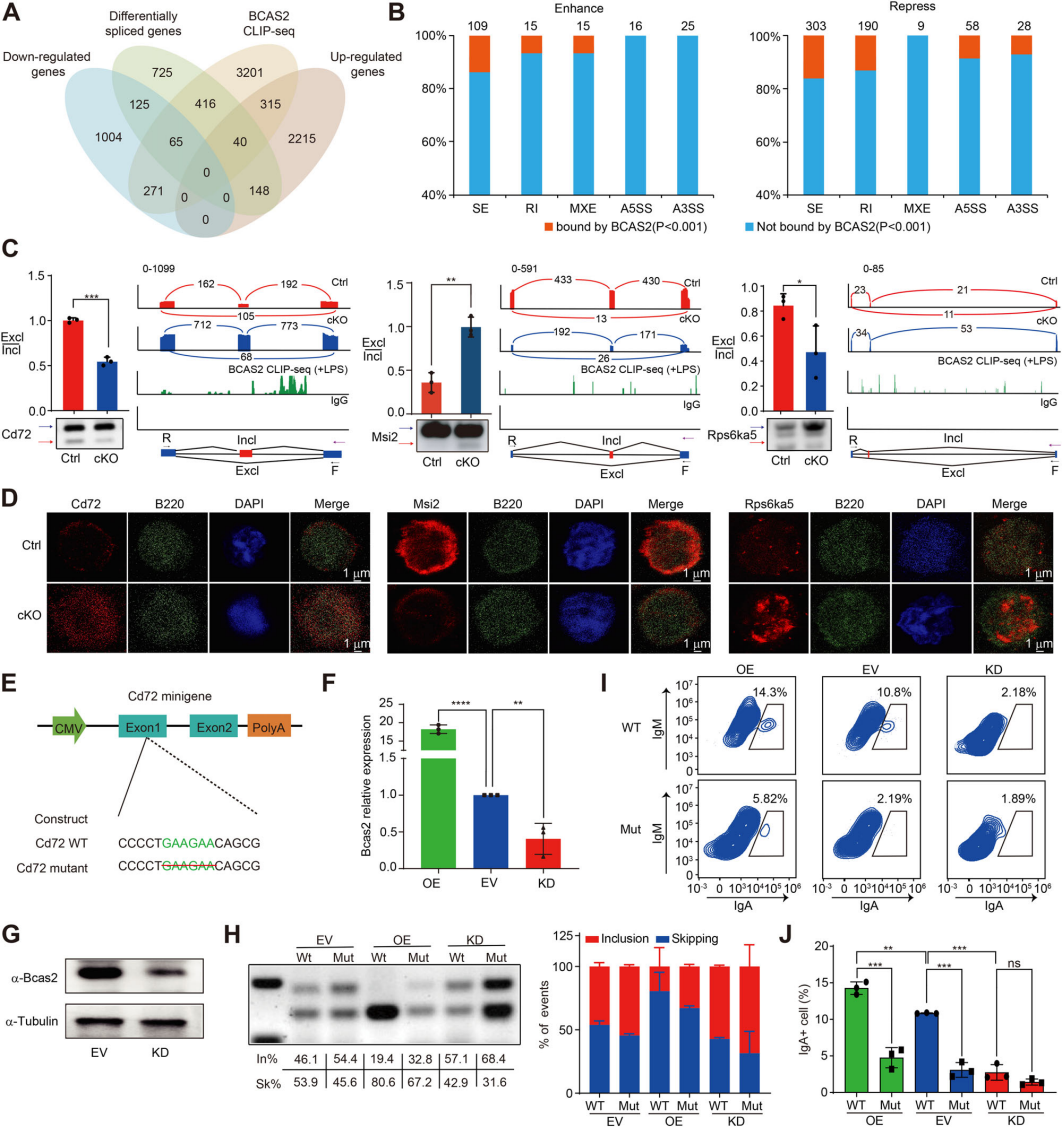
Bcas2 directly binds mRNA to regulate mRNA alternative splicing. (A) Venn diagram showing the correlation among down regulated, upregulated, alternatively spliced, and Bcas2‐binding genes. (B) Integrated RNA‐seq and CLIP‐seq analyses show that Bcas2 binds to genes undergoing either up‐ or down‐regulation of various types of alternative splicing events. (C) Alternative splicing of Cd72, Msi2 and Rps6ka5 in the spleen of LPS+IL4 stimulated WT and *Bcas2*‐cKO B cells was analyzed by RT‐PCR (*n* ≥ 3). The ratios of inclusion (Incl) to exclusion (Excl) are shown accordingly. Analysis of CSR‐associated, DNA recombination and repair‐associated, and Bcas2‐binding gene expression and exon‐exon junctions. The Bcas2‐binding peaks of meiosis‐related gene transcripts are shown. Purple arrow, direction of transcription. F, forward primers; R, reverse primers; D, Co‐immunostaining of Ctrl and cKO was performed using target protein (Cd72, Msi2 and Rps6ka5) and B220 antibodies from CH12 cell lines. DNA was stained with DAPI. Scale bar, 1 µm. (E) Schematic diagram of the construction of Cd72 targeted mutations (location of motifs significantly bound by Bcas2). (F) Quantitative PCR analysis of OE, EV and KD B cells. Bcas2 expression levels were normalized to gapdh transcripts and the EV group in quantitative PCR analysis (*n* = 3, mean ± SD). (G) Western blot analysis of EV and KD B cells on Bcas2 expression levels. (H) Alternative splicing of Cd72 in WT and mutant CH12 cell lines of OE, EV and KD was analyzed by RT‐PCR (*n* = 3). The ratios of inclusion (Incl) to exclusion (Excl) are shown accordingly. (I) Flow cytometric analysis for CSR in WT and mutant CH12 cell lines of OE, EV and KD after CIT stimulation. (J) Quantification of CSR shown in (I). Each symbol represents CH12 cell cultures (*n* = 3, mean ± SD). OE, overexpression; EV, empty vector; KD, knockdown.

A Venn diagram illuminated 65 downregulated and 40 upregulated genes among the 521 AS‐affected genes directly bound by Bcas2 (Figure 5A). In terms of AS events resulting in upregulation of gene expression, approximately 20% (109) of SE type, 10% (15) of RI type and 10% (15) of MXE type of AS events were mediated by Bcas2, while a very low proportion of events implicating Bcas2 in A5SS and A3SS (Figure 5B). By contrast, for AS events resulting in downregulation of gene expression, 303 of SE type, 190 of RI type, 58 of A5SS type, and 28 of A3SS type of AS events were governed by Bcas2 (Figure 5B). It is worthy to mention that SE was the primary type of AS where abnormal events frequently occurred, as shown before (Figure 3B). *Bcas2*‐cKO tended to result in increased SE type of AS events associated with altered gene expression, implicating an important role of Bcas2 in the regulation of gene expression through mediating AS.

We further determined the effects of Bcas2 on AS of *Cd72*, *Msi2*, and *Rps6ka5*. These selected genes have been shown previously to be implicated in CSR in B cells [45, 46]. In our study, *Bcas2*‐cKO significantly affected the AS of *Cd72*, *Msi2* and *Rps6ka5* in splenic B cells in response to LPS (Figure 5C). The presence of CD72, MSI2, and RPS6KA5 protein in B cells was validated (Figure 5D). To investigate the mechanism by which Bcas2 regulates the AS of *Cd72* and whether changes in *Cd72* AS directly affect B cell CSR, we mutated this gene by deleting the GAAGAA motif in its exon 1 (Figure 5E), which was shown to be the Bcas2 binding site (Figure 4E). Subsequently, we examined the AS of wildtype and mutant *Cd72* in CH12 cells transfected by empty vectors, *Bcas2* overexpression plasmid and *Bcas2* silencing RNA. We confirmed augmented gene expression of Bcas2 upon overexpression as well as diminished expression of Bcas2 on both transcriptional and protein levels in response to knockdown (Figure 5F,G). Following the endogenous splicing pattern, the proportion of exon exclusion in the CD72 minigenes was facilitated by the overexpression of Bcas2 compared to empty vectors. Transient knockdown of Bcas2 decreased the proportion of exclusion of the relative exons. Deleting the GAAGAA motif of CD72 significantly decreased Bcas2‐dependent exclusion (Figure 5H). In parallel, the population of IgM^−^ IgA^+^ cells dropped dramatically by *Cd72* mutation in the presence of Bcas2, but not in the absence of Bcas2 (Figure 5I,J). These results suggest that Bcas2 regulates the splicing of *Cd72* depending on the GAAGAA motif of *Cd72* transcripts, which further influences the antibody CSR in activated B cells.

### 2.7 The Bcas2‐Interacting Protein SRSF7 is Required for AS Regulation

**FIGURE 6:**
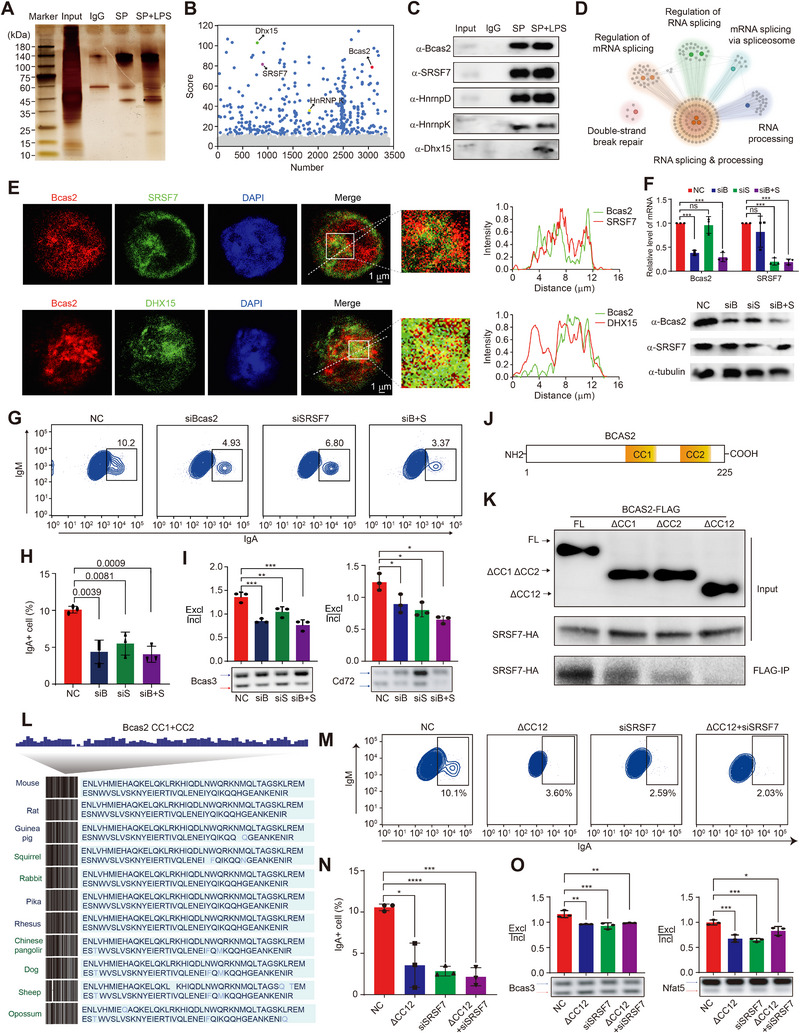
Bcas2 recruits AS‐related proteins to modulate AS in B cell. (A) Silver‐stained gel of Bcas2 and control immuno‐precipitates from splenic B cells. (B) Scatter plot of protein scores demonstrates significant differences in proteins between two groups of parallel mixed samples. (C) IP experiment was performed in spleen B cells extracts after LPS stimulation. (D) Network showing GO enrichment analyses of Bcas2‐binding proteins. (E) Co‐immunostaining and intensity scan map of CH12 cell was performed using Bcas2 and Bcas2‐binding proteins (SRSF7 and DHX15) antibodies. DNA was stained with DAPI. Scale bar, 1 µm. (F) Quantitative PCR analysis and western blot of B cell mRNA of NC, siBcas2, siSRSF7 and siS+B. Bcas2 and SRSF7 expression levels were normalized to gapdh transcripts and the NC group in quantitative PCR analysis (*n* = 3, mean ± SD). (G) Flow cytometric analysis for CSR in CH12 cell lines of NC, siBcas2, siSRSF7 and siS+B after CIT stimulation. (H) Quantification of CSR shown in (G). (I) Alternative splicing of Bcas3 and Cd72 in CH12 cell lines of NC, siBcas2, siSRSF7 and siS+B was analyzed by RT‐PCR (*n* = 3). The ratios of inclusion (Incl) to exclusion (Excl) are shown accordingly. (J) Model figure of CC1 and CC2 sequence location in Bcas2 protein. (K) Immunoprecipitation of Bcas2 flag and SRSF7‐HA. (L) Conservation of amino acid sequences in protein interaction regions across different species is demonstrated. (M) Flow cytometric analysis for CSR in CH12 cells with the deletion of CC1 and CC2 sequence of Bcas2 and SRSF7 knockdown. (N) Quantification of CSR shown in (M). Each symbol represents CH12 cell cultures (*n* = 3, mean ± SD). (O) The alternative splicing events in CH12 cells with the deletion of CC1 and CC2 sequence of Bcas2 and SRSF7 knockdown.

Previous studies have shown that Bcas2 is generally present as a component of protein complexes such as spliceosomes, in which the Bcas2‐interacting proteins may be required for Bcas2‐mediated AS. We next determined the proteins that interacted with Bcas2 that contribute to the regulation of AS in B cells through interactome proteomics analysis. We observed an increased abundance of proteins that interacted with Bcas2 after the splenic B cells were activated by LPS (Figure 6A and Figure S6A). We identified multiple AS‐related proteins, including SRSF7, HNRNP K, HNRNP D and DHX15, as Bcas2‐associated factors in the LPS‐activated splenic B cells by co‐immunoprecipitation and in activated CH12 cells with/without LPS by co‐localization staining (Figure 6B,C and Figure S6B). Interestingly, the interaction of Bcas2 with DHX15 was absent in splenic B cells without LPS stimulation ex vivo (Figure 6C). These results reveal the dynamics of interactome proteins of Bcas2 during the B cell activation. GO term enrichment analysis showed that the Bcas2‐interacting proteins were clustered in RNA splicing and processing, independent of LPS treatment (Figure 6D and Figure S6C). We depicted the interaction of Bcas2 with SRSF7, DHX15, and HNRNP K based on a hybrid strategy (Figure 6E and Figure S6D). We further examined the effects of SRSF7 on the antibody CSR mediated by Bcas2. First, we detected the decreased expression of Bcas2 and SRSF7 in the Bcas2 knockdown group and SRSF7 knockdown group, respectively (Figure 6F). The population of IgA^+^ cells was remarkably reduced in CH12 cells in the absence of Bcas2 or/and SRSF7 (Figure 6G,H). Likewise, suppressing the expression of Bcas2 and SRSF7 alone or together led to a decreased ratio of exclusion to inclusion in the Bcas3 and CD72 genes (Figure 6I). We also assessed the effect of another Bcas2‐interacting protein DHX15 and HNRNP K on CSR and found that silencing *Bcas2*, *Dhx15* and *Hnrnp K* alone or together significantly decreased the population of IgA^+^ cells in CH12 cells (Figure S6E–H). The alternative splicing events were also changed in each gene knockdown group (Figure S6I,J). To investigate the structural basis of Bcas2's interaction with SRSF7, we generated three plasmids within the pcDNA3.0 vector containing deletions of Bcas2's coiled‐coil (CC) domains—ΔCC1, ΔCC2, or both (Figure 6L). The sequence of the binding site was conservative across the species (Figure 6J). IP analysis using the FLAG tag in 293T cells revealed that SRSF7‐HA strongly bound to full‐length Bcas2, weakly bound to ΔCC1 and ΔCC2, and showed significantly reduced binding to ΔCC1CC2, indicating that both CC1 and CC2 sequences in Bcas2 are involved in binding to SRSF7 (Figure 6K). Transfection of CH12F3 cells with plasmids lacking both CCs, or SRSF7 siRNA, resulted in reduced CSR (Figure 6 M,N) and disrupted alternative splicing events (Figure 6O), highlighting the functional importance of these domains in CSR and splicing regulation. Altogether, the results suggest that the Bcas2‐interacting protein SRSF7 and DHX15 are required for the regulation of Bcas2‐mediated AS in B cells.

### 2.8 Bcas2‐Mediated as Plays a Role in Children With HIGM1

**FIGURE 7:**
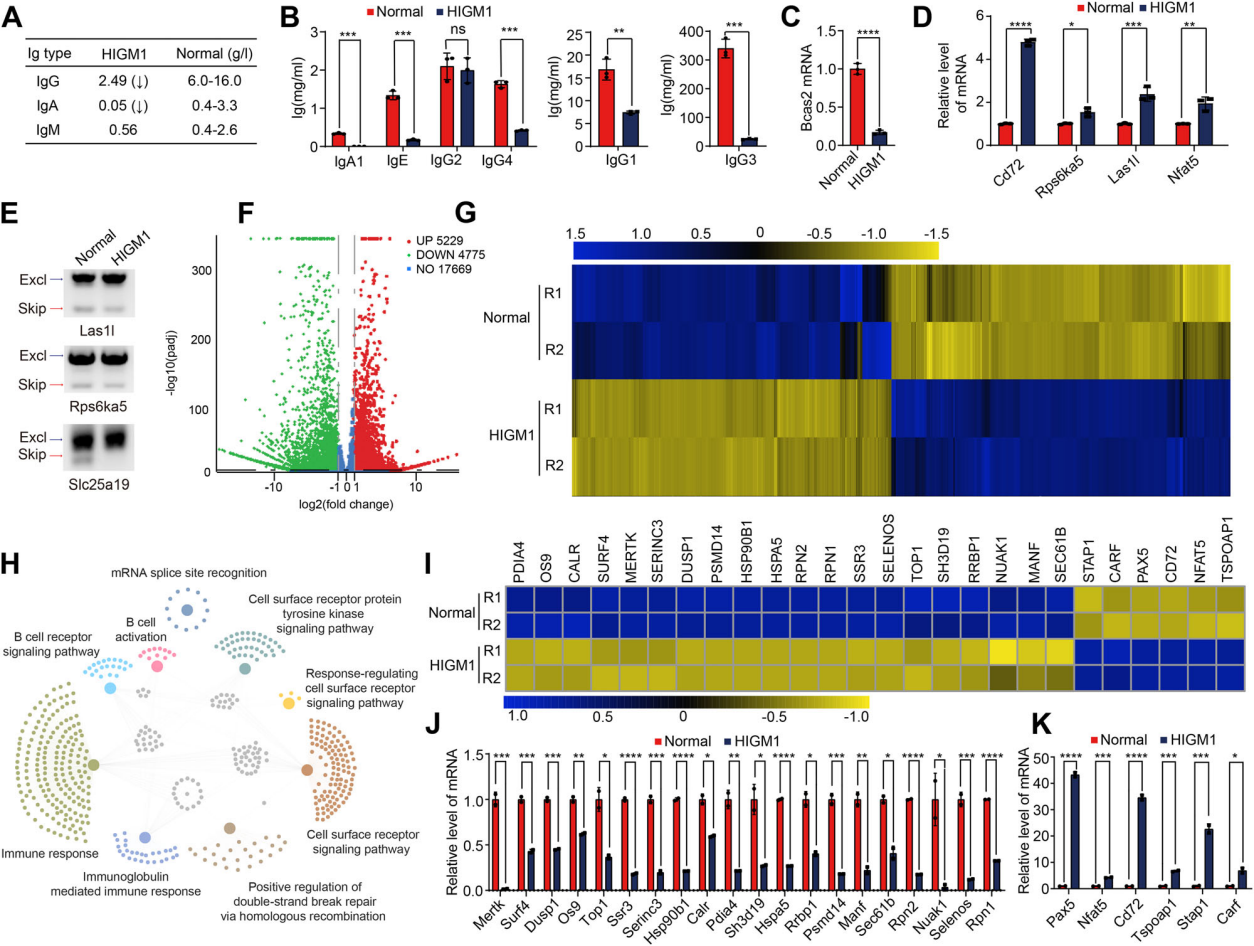
Bcas2 may be involved in the expression and alternative splicing of CSR‐related genes in hyper IgM. (A) Table presenting the concentrations of IgA, IgG, and IgM in the blood of the Hyper IgM patient alongside normal reference values. (B) Concentrations of IgG1, IgG2, IgG3, IgG4, IgA1, and IgE in Hyper IgM patient serum. Each symbol represents technical duplications and columns indicate the mean. (C) Bcas2 mRNA expression in normal people and HIGM1 patient. (D) Quantitative PCR analysis of B cell mRNA of normal human and Hyper IgM patient. Expression levels of genes altered after Bcas2 knockout (Cd72, Rps6ka5, Las1l, Nfat5) were normalized to gapdh transcripts and the normal human group in quantitative PCR analysis (*n* = 3, mean ± SD). (E) Alternative splicing of genes in Bcas2 knockout model was analyzed in normal human and Hyper IgM patient serum by RT‐PCR. (F) Volcano map displaying the distribution of differentially expressed genes from RNA‐seq data. The abscissa in the figure represents the gene fold change in the blood of normal human and Hyper IgM patient. |FoldChange| ≥ 1.5. Padj ≤ 0.05. Upregulated genes are shown as red dots, and downregulated genes are shown as green dots. (G) Cluster heatmap of differentially expressed genes. The abscissa is the genotype, and the ordinate is the normalized FPKM value of the differentially expressed gene. Blue indicates a higher expression level, while yellow indicates a lower expression level. (H) Network showing GO enrichment analyses of differentially expressed genes of Hyper IgM patient. (I) Cluster heatmap of several genes related to *Bcas2*‐cKO differentiated in RNA‐seq and the gene expression column reflecting the FPKM value. Blue indicates a higher expression level, while yellow indicates a lower expression level. (J,K) mRNA expression of upregulated and downregulated genes shown in (I).

Hyper‐IgM syndrome type 1 (HIGM1) is a rare primary immunodeficiency disease in which patients experience recurrent infections early in life [47, 48]. Studies have shown that patients with HIGM1 have significantly lower levels of IgA and IgG in the blood, while IgM levels are normal or elevated [47]. The genomic and proteomic lineages and pathogenesis of HIGM1 have not been elucidated. Here we reported a clinical case of HIGM1 in the children population, who was characterized by low levels of circulating IgG, IgA and IgE but the normal range of IgM levels (Figure 7A). Further analysis on the subclasses of IgA and IgG showed that the levels of IgA1, IgG1, IgG3 and IgG4, but not IgG2, in the serum of the HIGM1 patient were significantly lower than those in the age‐matched healthy individuals (Figure 7B). To explore the mechanisms underlying the regulation of circulating Ig in HIGM1, we performed serum proteomics and GO enrichment analysis based on the blood samples (Figure S8A‐I and Supplementary data Table S1). In total of 538 proteins in the serum of the HIGM1 patient were significantly altered in their levels compared with the healthy donor, of which 312 proteins were upregulated and 226 proteins were downregulated (Figure S8D). These proteins were enriched in the process related to B cell‐mediated immunity, Ig complex formation, Ig binding, and antigen‐binding (Figure S8F). Of note, there were significant decreases in the expression of 39 classes of antibody heavy chains including IGKV4‐1, IGHV3‐64D, IGLV8‐61, IGLL5, IGHV3‐15, IGKV3D‐15, IGV3‐10 in the HIGM1 patient (Figure S8G).

We then investigate whether the observed pathological changes in HIGM1 were associated with AS of antibody CSR. Interestingly, we identified that the expression of Bcas2 was significantly decreased in HIGM1 patient (Figure 7C). The Bcas2 target genes including *Cd72*, *Rps6ka5*, *Las1l*, and *Nfat5* were transcriptionally upregulated in the B cells of HIGM1 (Figure 7D), in line with the gene expression pattern in the murine activated *Bcas2*‐cKO B cells (Figure 2D). Besides, the AS of *Rps6ka5*, *Slc25a19* and *Las1l* was remarkably altered in HIGM1 (Figure 7E). To gain broader insight into molecular changes in HIGM1, we obtained the peripheral blood mononuclear cells (PBMCs) and performed transcriptomic and proteomic studies. According to transcriptomic analysis, 4775 genes were significantly downregulated on their transcriptional levels in the PBMCs of the HIGM1 patient compared to the control cohort (Figure 7F,G). GO enrichment analysis revealed that the genes that were transcriptionally altered were mainly enriched in the pathways related to double‐strand break repair, mRNA splicing, immune response, and B cell activation (Figure 7H). The Bcas2‐targeting genes including *Rpn1*, and *Top1* were downregulated while *Cd72*, *Nfat5* were upregulated in HIGM1 patient (Figure 7I,J), The expression of *Cd72* exons was markedly enhanced compared with the healthy individual (Figure S7A). Through Sanger sequencing of genomic DNA from the blood of the HIGM1 patient, we found a T to G mutation in the 3’UTR of Bcas2 DNA (Figure S7B), indicating that the mutation contributes to the instability of Bcas2 transcript. We then overexpressed the wild‐type Bcas2 gene and Bcas2 mutant (Bcas2‐Mut) gene carried by plasmids in the CH12 cell line. Mutations in the 3' UTR significantly altered the stability of *Bcas2* mRNA (Figure S7C). We observed that the splicing changes in *Bcas3* and *Nfat5* in the CH12 cell lines with Bcas2 mutated, were similar to those seen in *Bcas2* knockout CH12 cells (Figure S7D). Moreover, the antibody production capacity of the CH12 cell line with the mutated *Bcas2* gene was also significantly reduced, mirroring the effects observed with *Bcas2* knockdown in the CH12 cell line (Figure S7E,F). Collectively, our data reveal that Bcas2 may regulate AS in the pathogenesis of HIGM1.

**FIGURE 8:**
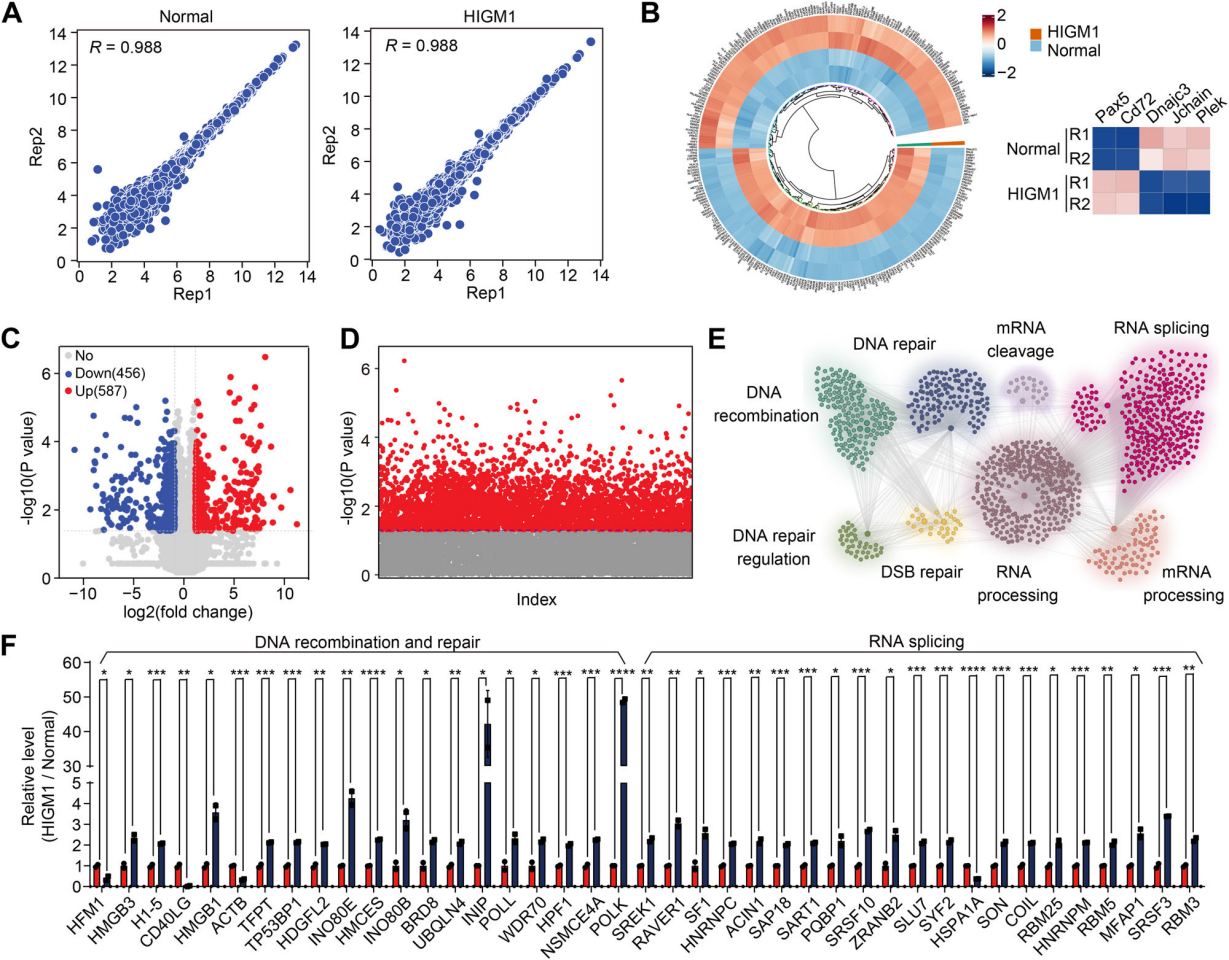
Bcas2 may be involved in the expression and alternative splicing of CSR‐related genes in hyper IgM. (A) Pearson's correlation analysis shows the coefficient between two replicates in the sample of the normal people and the patient in PBMC proteomics. (B) Circos representing the correlation between different proteins and normal people or the HIGM1 patient; with a cluster heatmap of several proteins related to antibody formation. Red indicates a higher expression level, while blue indicates a lower expression level. (C) Volcano map displaying the distribution of differentially expressed proteins from proteomics data. The abscissa in the figure represents the protein fold change in PBMC of normal human and Hyper IgM patient. |FoldChange| ≥ 1.5. Padj ≤ 0.05. The ordinate indicates the significance of gene expression differences between the HIGM patient and normal subjects. Upregulated genes are shown as red dots, and downregulated genes are shown as blue dots. (D) Scatter plot of differentially expressed proteins. The abscissa is the protein indexes, and the ordinate is the −log10 (*p* value) of the differentially expressed protein. Red indicates a higher expression level above the threshold with the −log10 (*p* value) equals 1.5. (E) Network showing GO enrichment analyses of differentially expressed proteins of Hyper IgM patient. (F) Differentially expressed proteins of Hyper IgM patient from PBMC proteomics. All the relative expression levels of proteins in normal people were normalized to 1.

To explore the protein networks in HIGM1 patients, proteomic results were performed and technically reproducible (Figure 8A). The expressions of 587 proteins including PAX5 and CD72 were significantly elevated in the HIGM1 patient compared to the healthy individual, while the levels of 456 proteins including DNAJC3, J chain, and PLEK were significantly decreased (Figure 8B–D). Through GO enrichment the proteins were clustered in the process involving DNA repair, DNA recombination, RNA splicing, and RNA processing (Figure 8E). The significantly changed proteins involved in DNA recombination and repair and RNA splicing were displayed (Figure 8F). The results of AS, transcriptomics and proteomics in the blood of HIGM1 patients were reminiscent of the findings of AS events related to antibody CSR in the splenic B cells of *Bcas2*‐cKO mice (Figures 2G and 7H, Figures 5C and 7I). Together, our data suggest that Bcas2 was implicated in the pathology of HIGM1 for it affects the AS of CSR‐related genes in HIGM1 patient.

## 3 Discussion

The present study reveals a mechanism during B cell activation and antibody production by which Bcas2 affects the antibody CSR through mediating AS and translation of proteins that govern the post‐switched transcription of the V(D)J of antibodies. More specifically, Bcas2 interacts with SRSF7 at a conservative circular domain, forming a complex to regulate the AS of genes that are engaged in the antibody CSR, leading to broad‐spectrum alterations in antibody production in activated B cells.

Bcas2 has been acknowledged as an RBP that plays a role in AS during mitosis in highly proliferating cells such as sperm cells and cancer cells, with little information available on its function in B cells. Here, we demonstrate the ubiquitous expression of Bcas2 in various types of B cells and immune tissues. More importantly, our study shows that Bcas2 was transcriptionally increased by about sevenfold in response to LPS while the antibody class conversion was disrupted due to deletion of *Bcas2* in activated B cells. These findings inspire us to focus on the regulation of CSR through Bcas2‐mediated AS. By contrast, studies have shown that YTHDC1, another RBP that acts as m6A reader, can induce CSR by promoting 3' end processing of long non‐coding RNAs (SµGLT) that facilitated CSR at the IgH locus in B cells [49]. Similarly, the RBP DDX1 was shown to regulate CSR at the IgH locus, which are associated with the transition between the S region R‐loop structure and G4 [16]. Another viewpoint suggests that E3‐ubiquitin ligase Nedd4 can regulate transcriptional pause by forming complexes with AID [50]. Nevertheless, studies rarely genome‐wide elucidate how RBP regulates CSR through AS. In this study, we focus on demonstrating the role of Bcas2 in the activated B cells in GC tissues during the immune response.

**FIGURE 9:**
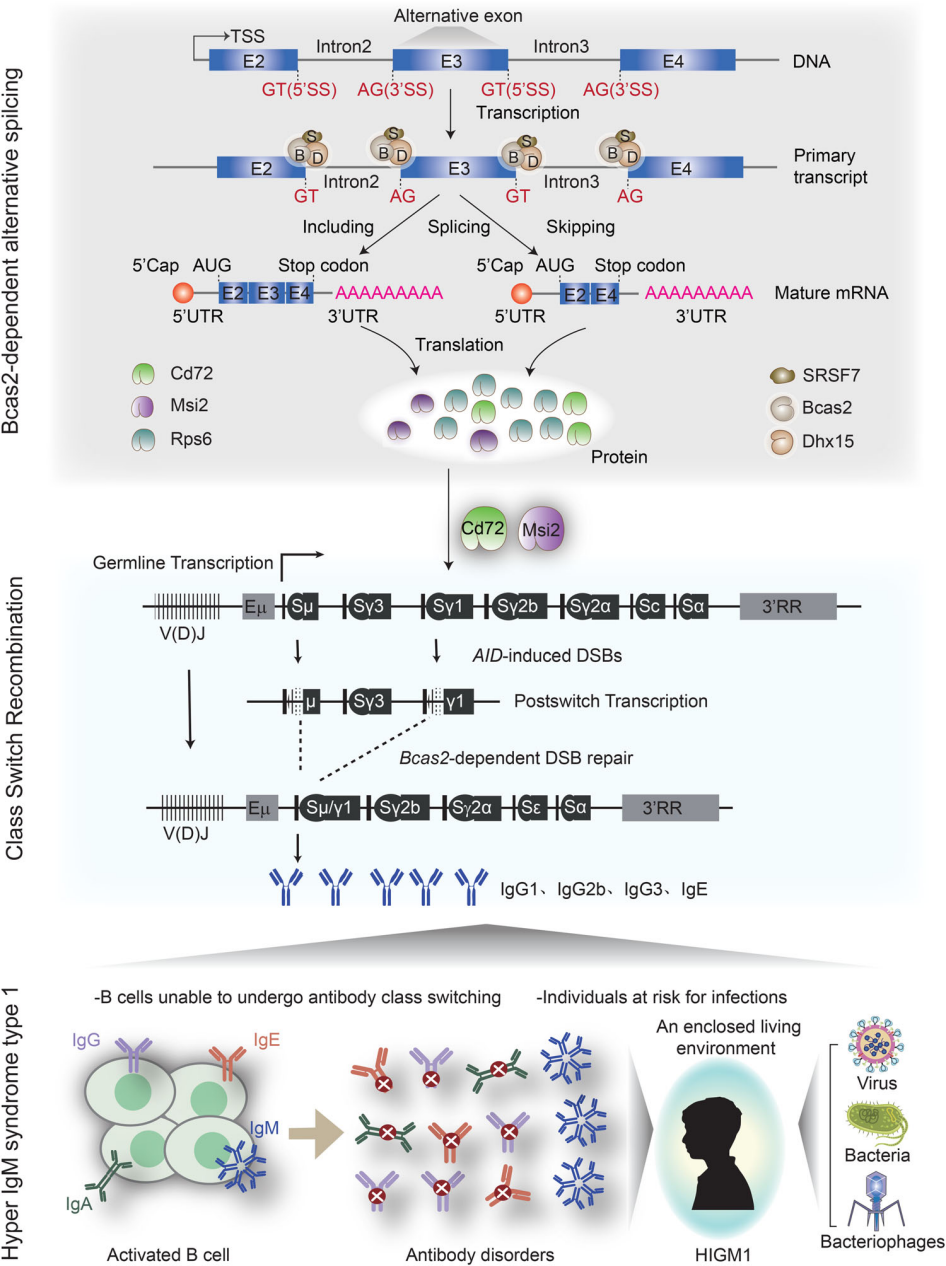
Model for Bcas2 Role in CSR. Bcas2 recruits DHX15 and SRSF7 to form a protein complex for the splicing of CSR‐related mRNA at the 5' ss and 3' ss, generating two mature mRNA isoforms, which are ultimately translated into CSR‐related proteins. These CSR‐related proteins bind to DNA associated with antibodies, affecting the DSB repair of the relevant DNA after AID induction, thereby influencing the transcription of antibody isotypes and regulating the production of IgG1, IgG2b, IgG3, and IgE. Finally, through the RNA and protein level analysis of blood samples from patients with type I Hyper IgM syndrome, it is speculated that Bcas2 deficiency should affect alternative splicing, thereby regulating antibody CSR in the patient's blood.

In the present study, Bcas2 was shown to have an influence on the AS of a variety of genes involved in AS, including *Msi2*, *Cd72* and *Rps6ka5*. More importantly, we identified the binding motif of Bcas2 on the RNAs and revealed its important role in the regulation of Bcas2‐dependent AS and CSR. These findings provide additional information for a better understanding of Bcas2's function [28, 30, 31] and the underlying mechanisms of Bcas2 regulation in diseases [32]. It has been shown that MSI2, RPS6KA5 and CD72 can regulate post‐switched transcription of the V(D)J of antibodies by binding to S regions [51, 52, 53]. We also identified the Bcas2‐interacting proteins in murine splenic B cells and CH12 cell line, among which SRSF7, HnRNP K and DHX15 turned out to be splicing factors in the previous studies [54, 55, 56]. We showed that the concurrence of Bcas2 and its interacting proteins is crucial for AS and CSR in activated B cells. In this sense, our study provides insights into the mechanism of how Bcas2 modulates the AS of CSR‐related genes (Figure 9).

It has been shown that RBP can govern the fate of mRNA through regulating the RNA coding via binding to specific motifs with lengths of 4–8 nt. The high selectivity of RBP to RNA coding confers the functional specificity of RBP in various scenarios [57, 58]. Associations between different RBPs usually result in the formation of various macromolecular complexes, in which each RBP may contribute to the recognition of specific RNA codes. Such complexes incorporating multiple RBPs create complicated endogenous specificities for a variety of RNA codes [59]. Previous studies have established a DNA loading model mediated by Spt5‐, 14‐3‐3‐ and RPA in the process of CSR [60, 61, 62, 63]. By contrast, we intent to elucidate the target specificity of Bcas2 based on RNA mediated recruitment model. Firstly, the high selectivity of Bcas2 to the specific motif of RNA confers the selective regulation of its target genes. Secondly, the binding domain of Bcas2 with the splicing factors is evolutionarily conservative across species. A possible biological significance could be that the selectivity of the complex can be preserved at best. HIGM1 is a rare immunodeficiency disease in which patients experience recurrent infections early in life [47, 48]. B cells play an important role in the development of HIGM1 considering the fact that activated B cells are the unique site for antibody production. To date, the pathogenesis of HIGM1 has not been elucidated yet. Based on high‐throughput analysis of the genomic and proteomic lineages in the HIGM1 patient's blood, we for the first time have shown the AS events and CSR in HIGM1. Intriguingly, the AS pattern and serum Ig profile in the HIGM1 patient match well with those observed in the *Bcas2*‐cKO mice in this study. This encouraged us to link Bcas2 with etiology of HIGM1. Indeed, we identified the mutation in the 3’UTR of *Bcas2* gene in HIGM1. Mutation at the 3’UTR site of the genome may lead to instability of mRNA, thus decreasing the gene expression and protein level. It is conceivable that the function of Bcas2 is impaired and Bcas2‐mediated AS may be disrupted in HIGM1. From this perspective, our study suggests that Bcas2 serves as a potential factor in the pathogenesis of HIGM1. Further research can focus on establishing the link between different types of AS and HIGM1.

Overall, we for the first time elucidate the mechanism of how Bcas2‐mediated AS affects the antibody CSR during the B cell activation. Besides, the target genes of Bcas2 in B cells have been validated, which may contribute to the drug development. Our study reveals that AS of genes that are important for CSR can eventually affect the diversity of antibody [64]. Significantly, we for the first time have demonstrated that the serum antibody profile is highly informative in children with HIGM1 and revealed the alternative splicing diversity associated with Bcas2. Therefore, our study may provide new insights into therapeutic strategies for immunodeficiency diseases and autoimmune diseases in the future.

## 4 Materials and Methods

### 4.1 Patient Samples Collection

The HIGM1 patient and the normal subjects enrolled for genetic analysis had written informed consent, and the protocol was approved by the Ethics Committee of the Chinese People's Liberation Army (PLA) General Hospital. The studies are strictly in compliance with the Declaration of Helsinki. The blood samples from subjects were used for RNA‐seq analysis, and the serum samples were used to detect antibody levels by ELISA. PBMC and serum proteomics were analyzed through DIA mass spectrometry.

### 4.2 Animal Breeding and Genotyping

C57BL/6N and ICR mice were purchased from Beijing Vital River Laboratory Animal Technology Co., Ltd. *Bcas2* ^fl/+^ mice were generated, bred and kindly provided by Professor Lei Li at the Chinese Academy of Sciences. AID‐Cre mice were purchased from the Jackson Laboratory [65]. The *Bcas2*‐cKO mice were generated by backcrossing AID‐Cre mice with *Bcas2* ^fl/fl^ mice derived from parental *Bcas2* ^fl/+^ mice. Genotyping for *Bcas2* ^fl/+^, AID‐Cre and *Bcas2* ^fl/fl^ mice was conducted as previously described [33, 66]. The primers for genotyping are summarized in supplementary data Table S2. Mice were group housed and kept in a temperature‐controlled environment (22°C ± 2°C) with a 12 h:12 h light‐dark cycle in specific pathogen‐free (SPF) conditions. All the animal experiments in this study were performed and adhered to the guidelines with the approval of the Institutional Animal Care and Use Committee of China Agricultural University (Approval No. AW02504202‐5‐1).

SPF male C57 mice aged about 7 weeks were procured from Beijing HFK Bioscience Co., Ltd, located in Beijing, China. Throughout the duration of the experiment, the mice had unrestricted access to sterilized water and standard rodent chow sourced from the China Agricultural University, Beijing, China. All animal‐related procedures were conducted according to the Guidelines for the Care and Use of Animals. The study's protocol underwent rigorous scrutiny and received approval from the Institutional Animal Care and Use Committee of China Agricultural University, Beijing, China (Approval No. AW02504202‐5‐1).

### 4.3 Immunization of OVA and NP‐KLH

7–8 weeks old C57BL6/J male mice were procured from Beijing HFK Bioscience Co., Ltd, located in Beijing, China. Immunization of OVA and that of NP‐KLH were performed as described before [67, 68, 69]. In brief, mice were subcutaneously immunized with in the flank with 100 µg of endotoxin‐depleted 2 mg mL^−1^ OVA (Catalog#HY‐P0286, MCE) and 1 mg mL^−1^ NP‐KLH (Catalog#N‐5060‐5, Biosearchtech), which was formulated in Complete Freund's adjuvant (Sigma‐Aldrich) at a 1:1 ratio. Mice received a single dose of each stimuli on a total volume of 100 µL via intraperitoneal injection. Two weeks later, the mice were euthanized and blood samples were collected. Serums were obtained after 4°C, 3000 × *g* centrifuge for 10 min and subsequently aliquoted and stored at −80°C.

### 4.4 Cell Culture

Primary B cells were isolated freshly from the 8‐week‐old *Bcas2*‐cKO mice and their control littermates. CH12F3 cells and murine splenic B cells were cultured in RPMI 1640 medium (Catalog#sh30809.01, Hyclone), supplemented with 5% fetal bovine serum (Catalog#P30‐3302, PAN), L‐glutamine (Catalog#25030081, Thermo), sodium pyruvate (Catalog#11360070, Thermo) and penicillin‐streptomycin antibiotics (Catalog#15070063, Thermo). 293T cells were cultured in DMEM medium (Catalog# 12430054, Gibco), supplemented with 10% fetal bovine serum (Catalog#P30‐3302, PAN) and penicillin‐streptomycin antibiotics (Catalog#15070063, Thermo). Cells were maintained in a humidified incubator set at 37°C with 5% CO2.

### 4.5 Cell Proliferation Assay

Approximately 1 × 10^7^ cells were harvested and incubated in 2 mL of staining buffer containing 2.5 µM carboxyfluorescein succinimidyl ester (CFSE, Cat#423801, Biolegend) and 0.1% BSA in phosphate‐buffered saline (PBS) at 37°C for 10 min without light [70]. The staining was terminated using five times the original staining volume of RPMI‐1640 medium containing 10% FBS, after which cells were washed three times and cultured in RPMI‐1640 medium for 3 days. Cells were captured before seeding and on Day 3 using a fluorescence microscopy and flow cytometry instrument, and the cell number was analyzed using Image J and Flowjo program.

### 4.6 Cell Transfection Assay

Transient knockdown of *Bcas2* genes in CH12F3 cell lines was achieved via their siRNA. In brief, cells were seeded at a density of 1 × 10^6^ cells/mL. 1 nM of siRNA in the electroporation mixture (Catalog#MPK10025, Thermo) were added to the cells, followed by transfection with electroporation using Neon Transfection Device (Invitrogen). Subsequently, cells were cultured at a density of 1 × 10^5^ cells/mL in the presence of 1 µg mL^−1^ purified anti‐mouse/rat CD40 (Catalog#16‐0401‐82, Invitrogen), 10 ng mL^−1^ recombinant murine IL‐4 (Catalog#214‐14, Peprotech) and 2 ng mL^−1^ recombinant human TGF‐β1 (Catalog#315‐15, Peprotech) for 3 days [71]. Transient knockdown efficiency was validated through qRT‐PCR. Bcas2 siRNA were synthesized by Guangzhou RiboBio Co., Ltd. according to the designed sequences CAGCTTACAGCTGGATCTA.

For the transfection of 293T cells, GeneTwin gene transfection reagent (Biomed, China) was used. A total of 6 µg of plasmid DNA was dissolved in 100 µL of PBS. Then 18 µL of the transfection reagent was mixed with PBS to create a final volume of 100 µL. This mixture was combined with the plasmid solution to reach 200 µL and incubated for 20 min. The transfection complex was then added to 293T cell cultures at ≈70% confluency, mixed gently, and incubated for 48 h.

### 4.7 Dual‐Luciferase Reporter Assay

The wild‐type and 3'UTR mutant fragments of the mouse Bcas2 gene were cloned into the PsiCHECK‐2 vector (synthesized by BGI). The plasmids were then electroporated into CH12 cells and cultured for 48 h, followed by the measurement of luciferase activity according to the instructions of the assay kit (Promega, Catalog#E1960).

### 4.8 B Cell Activation

Mouse splenic B cells were isolated using B cell isolation kits according to the manufacturer instructions (Catalog#19854, Stem cell). Cells were washed three times with 1 × PBS and cultured at a density of 3 × 10^5^ cells/mL. Then, cells were exposed to 25 µg mL^−1^ of LPS (Catalog#L3024‐10MG, Sigma‐Aldrich) or LPS plus 10 ng mL^−1^ of recombinant murine IL‐4 (Catalog#214‐14, Peprotech) for 3 days.

### 4.9 qRT‐PCR

Total RNA extraction was done using RNAiso Plus (Catalog#9109, Takara) and quantified on a NanoDrop One Spectrophotometer (Thermo scientific). cDNA synthesis was conducted by following the instructions provided with the HiScript kit (Catalog#Q711, Vazyme, China). The gene expression was determined using QuantStudio 5 Real‐Time PCR instrument (384‐Well Block) (Applied Biosystems). AS analysis was performed using a PCR amplification instrument (Eppendorf), after which the PCR products were subject to agarose gels and visualized via a Gel image analysis system (Tanon 1600, China). The relative expression of target genes was normalized using *Gapdh* or *Actb* as the reference gene. The primers for qRT‐PCR and AS analysis were synthesized by Sangon Biotech and their sequences were listed in Supplementary data Table S2.

### 4.10 RNA‐Sequencing Analysis

Total RNA was extracted from mouse splenic B cells treated with LPS+IL4, as described above. mRNA was enriched using poly‐T oligo‐attached magnetic beads. The purified mRNA was subject to fragmentation to establish a transcriptome sequencing library. By using a TruSeq PE Cluster kit v3‐cBot‐HS (Illumina) on a cBot Cluster Generation System, index‐coded samples were clustered in accordance with the manufacturer's guidelines. Then, the library preparations were sequenced on the Illumina NovaSeq platform that generated 150 bp paired‐end reads. Rigorous quality control measures were implemented post‐sequencing. A reference genome index was established, and paired‐end clean reads were aligned to this reference genome using HISAT2 software (version 2.0.5). FeatureCounts (version 1.5.0) was then employed to enumerate reads mapped to each gene. Following this, an FPKM value of each gene was calculated, factoring in both the gene's length and the read count mapped to it. Differential gene expression between *Bcas2*‐cKO and control mice (comprising a biological replicate per condition) was conducted using the DESeq2 R package (version 1.20.0) [72]. Genes demonstrating a padj value ≤0.05, as identified by DESeq2, were delineated as differentially expressed.

### 4.11 CLIP‐seq Analysis

CLIP‐sequencing analysis was performed as described previously [73], with minor modifications as follows. Briefly, mouse splenic B cells were isolated from C57BL/6J mice and purified using the B cell isolation kit (Catalog#19854, Stem cell). The cells were treated with LPS for 3 days, after which cells were washed using 1 × PBS supplemented with 2% bovine serum albumin (BSA). The cells were exposed to ultraviolet light for cross‐linking RNA molecules with their binding proteins. Cell lysates were obtained, followed by immunoprecipitation (IP) of RNA‐protein complexes using antibodies specifically targeting the protein of interest.

Then, the IP samples underwent complete washing to remove non‐specifically bound RNA molecules. RNA fragments were then liberated from the protein‐RNA complexes and prepared for high‐throughput sequencing library construction using the NEBNext Ultra II RNA Library Prep Kit.

Qubit fluorometric quantitation and the Agilent Bioanalyzer were applied to ensure the quantification and quality of the sequencing libraries. Subsequently, the libraries were subjected to sequencing on an Illumina NextSeq 500 platform to generate either single‐end or paired‐end reads. Raw sequencing data underwent processing to eliminate adaptor sequences and low‐quality reads using Cutadapt and Trimmomatic software, respectively. The processed reads were aligned to the reference genome using the STAR aligner, with uniquely mapped reads retained. Homer (version 4.9.1) was used for peak annotation based on the mm10 genome assembly, as well as for the analysis of Bcas2‐binding motifs. The quality of the replicates was evaluated by calculating the pairwise Spearman correlation coefficients.

### 4.12 Plasmid Construction

The pcDNA3.0‐Bcas2 plasmid with an ampicillin resistance gene was designed to contain the CMV promoter upstream of the Bcas2 coding sequence, followed by a bovine growth hormone polyadenylation signal (polyA). The pcDNA3.0‐Bcas2 vector backbone was linearized by restriction enzyme digestion with two enzymes (EcoR I and Kpn I) to create compatible ends for ligation with the PCR‐amplified Bcas2 fragment. After T4 DNA ligase to create the target plasmid, the ligated plasmid was transformed into competent *E. coli* cells which were cultured on LB agar plates containing ampicillin for selection of positive transformation.

The plasmid extraction kit (Catalog#AG21032, Accurate Biology, China) was applied for plasmid extraction according to the manufacturer's instructions.

### 4.13 Flow Cytometry

Flow cytometry analysis was performed using a BD LSRFortessa flow cytometer (Becton, Dickinson and Company, US). Prior to analysis, cells were resuspended in PBS supplemented with 1% bovine serum albumin (BSA) to a final concentration of 1×10^6^ cells/mL.

For the immunophenotyping experiments, cells underwent staining with a combination of fluorescently labeled antibodies targeting cell surface markers (B220, Catalog#103224, Biolegend; CD19, Catalog#152418, Biolegend; IgD, Catalog#405711, Biolegend; IgM, Catalog#406511, Biolegend; CD43, Catalog#143203, Biolegend; CD25, Catalog#113703, Biolegend; CD5, Catalog#100641, Biolegend; CD23, Catalog#101607, Biolegend; CD93, Catalog#136505, Biolegend). Initially, 1 × 10^6^ cells were distributed into 5 mL polystyrene tubes, where cells were exposed to the appropriate antibody mixture for 30 min at 4°C in the absence of light. Following this incubation period, cells underwent two washes with PBS before being suspended in 300 µL of PBS‐BSA solution.

In the RNA silencing experiment, B220 (Catalog#103224, Biolegend), CD19 (Catalog#152418, Biolegend), IgM (Catalog#406511, Biolegend), and IgA (Catalog#407003, Biolegend) were introduced into the cellular mixture at a concentration ratio of 1:400. As for the cytokine induction, cells co‐cultured with LPS were labeled with B220, CD19, IgG2b (Catalog#406705, Biolegend), IgG3 (Catalog#406803, Biolegend) with the secondary antibody (Catalog#405203, Biolegend) at a uniform ratio of 1:400 for each antibody. Conversely, cells co‐cultured with a combination of LPS and IL‐4 were labeled with B220, CD19, IgG1 (Catalog#406605, Biolegend), and IgE (Catalog#406907, Biolegend) at the ratio of 1:400 for each antibody. For in vivo immunization, splenic B cells were collected and then labeled with CD95 (Catalog#152606, Biolegend), GL7 (Catalog#144614, Biolegend), CD138 (Catalog#142509, Biolegend), CD38 (Catalog#102741, Biolegend), IgD (Catalog#405727, Biolegend). at the ratio of 1:400 for each antibody. Before the cells were labeled, each cellular mixture was incubated with CD16/CD32 antibody at a uniform ratio of 1:400 (Catalog#BE0307‐1 mg, BioXcell).

Data acquisition utilized BD FACSDiva software version 8.0.1, while data analysis was conducted using FlowJo software version 10.7.1. Gating strategies were employed to exclude debris, aggregates, and non‐viable cells based on forward scatter (FSC), side scatter (SSC), and PI staining. Compensation for fluorescence was carried out using single‐stained controls for each fluorochrome.

### 4.14 Western Blotting

Total protein was extracted using cell lysis buffer (P0013, Beyotime), supplemented with PMSF (1:100, Catalog#ST506, Beyotime), and a protease inhibitor cocktail (1:100, Catalog#P1005, Beyotime). The concentration of protein was determined using the Pierce BCA Protein Assay Kit (Catalog#23227, ThermoFisher). Subsequently, the protein lysates were separated via sodium dodecyl sulfate‐polyacrylamide gel electrophoresis and transferred to polyvinylidene fluoride membranes (Catalog#IPVH00010, Millipore). Following this, the membranes were blocked with 5% BSA for 2 h and incubated overnight at 4°C with primary antibodies (Beta actin, Catalog#66009‐1‐Ig, Proteintech; Bcas2, Catalog#PA2466, Abmart; Tubulin, Catalog#11224‐1‐AP, Proteintech; DHX15, Catalog#82137‐1‐RR, Proteintech; Srsf7, Catalog#PA3317S, Abmart; HNRNP K, Catalog#67708‐1‐Ig, Proteintech; HNRNP D, Catalog#ab259895, Abcam; AID, Catalog#4975S, CST). The membranes were then incubated with secondary antibodies (Goat anti‐rabbit IgG (H+L), Catalog#31460, Invitrogen; Goat anti‐Mouse IgG (H+L), Catalog#31430, Invitrogen) at room temperature for 2 h. Protein visualization was carried out using a Tanon 5200 chemiluminescence imaging system following incubation with ECL Plus (Catalog#PE0010, Solarbio).

### 4.15 Immunofluorescence Staining

Immunofluorescence staining was performed for Bcas2 (Catalog#AB108330, Abcam), Srsf7 (Catalog#sc‐390126, Santa), HNRNP K (Catalog#67708‐1‐Ig, Proteintech), DHX15 (Catalog#sc‐271686, Santa), MSI2 (Catalog#T55629S, Abmart), CD72 (Catalog#PH4955S, Abmart), RPS6KA5 (Catalog#PA6507S, Abmart) proteins in CH12 cells. Cells were washed with PBS and then fixed with 4% paraformaldehyde (PFA) in PBS for 15 min at RT. Cells were incubated with 0.1% Triton X‐100 in PBS for 5–10 min at RT. Wash cells three times with PBS to remove Triton X‐100. Block nonspecific binding sites by incubating cells with blocking solution (Catalog#P0260, Beyotime) for 1 h at RT. Samples were incubated with the primary antibody overnight at 4°C. Wash cells three times with PBS and then incubate cells with the secondary antibody solution (anti‐rabbit antibody, Catalog#P0183, Beyotime; anti‐mouse antibody, Catalog#P0196, Beyotime) at a uniform ratio of 1:400 for 1 h at RT in the dark. Mount coverslips with cells onto glass slides using mounting medium (Catalog#P0265, Beyotime). Snap the photos using confocal microscopy (Zeiss LSM 900, German).

### 4.16 Co‐Immunoprecipitation (co‐IP) and Silver Stains

Total protein was extracted utilizing cell lysis buffer (P0013, Beyotime), supplemented with PMSF (1:100, Catalog#ST506, Beyotime), and a protease inhibitor cocktail (1:100, Catalog#P1005, Beyotime). Following 20 min incubation on ice, the lysate underwent pre‐clearing with 10 µL of protein A/G beads (Catalog#80104G, Invitrogen) at 4°C for 1 h. Subsequently, 5 µg of Bcas2 antibody (Catalog#PA2466, Abmart) and normal mouse IgG (Catalog#2729S, CST) were added to the lysate. The mixture was incubated overnight at 4°C. The next day, the lysate was incubated with 50 µL of protein A/G beads on a head‐over rotator at 4°C for 4 h. The agarose complexes containing antibodies and target proteins were washed, eluted and subjected to western blotting. The intricate structure of proteins underwent examination at the protein mass spectrometry facility through immunoprecipitation‐mass spectrometry (IP‐MS) employing a Thermo Q‐Exactive high‐resolution mass spectrometer (Thermo Scientific, Waltham, MA, Qinghua University). Subsequently, the raw data acquired from the mass spectrometer were subject to preprocessing using Mascot Distiller 2.4 for peak identification. The resultant peak lists were subsequently interrogated against the UniProt mouse database using Mascot 2.5 search engine. For silver stains, Pierce Silver Stain Kit Thermo Scientific was used according to its instruction [74].

### 4.17 GO Enrichment Analysis

The GO enrichment analysis for differentially expressed genes and AS genes was conducted using the clusterProfiler R package (version 3.4.4). To correct for gene length bias, the genes that presented a total TPM sum across all samples above 1 were included in the background list. The mouse reference genome GRCm38/mm10 was utilized for the analysis. The Benjamini–Hochberg method was employed to adjust for multiple testing. Enrichment analysis with corrected *p*‐values below 0.05 was considered to significantly enrich differentially expressed genes and AS genes.

### 4.18 DIA Mass Spectrometry Data Acquisition

For each sample, peptides were separated using the Vanquish Neo UHPLC system (Thermo Scientific). The chromatographic separation was performed with buffer A (0.1% formic acid in water) and buffer B (0.1% formic acid in acetonitrile, 80% acetonitrile). The column was equilibrated with 96% buffer A. Inject the sample into the Trap Column (PepMap Neo 5 µm C18 300 µm × 5 mm, Thermo Scientific) and perform gradient separation using the analytical column (µPAC Neo High Throughput column, Thermo Scientific). The liquid phase gradient was set as follows: 0–0.1 min: Linear gradient of buffer B from 4% to 6%; 0.1–1.1 min: Linear gradient of buffer B from 6% to 12%; 1.1–4.3 min: Linear gradient of buffer B from 12% to 22.5%; 4.3–6.1 min: Linear gradient of buffer B from 22.5% to 45%; 6.1–8 min: Buffer B maintained at 99%. Peptides were then analyzed using an Orbitrap Astral mass spectrometer (Thermo Scientific) with DIA (Data Independent Acquisition). The analysis duration was 8 min with the following parameters: Electrospray voltage: 2.2 kV; detection mode: positive ion; precursor ion scan range: 380–980 *m*/*z*; first MS resolution: 240,000; AGC target: 500%; first MS maximum IT: 3 ms; second MS resolution: 80,000; AGC target: 500%; second MS maximum IT: 3 ms; RF‐lens: 40%; MS2 activation type: HCD; isolation window: 2 Th; normalized collision energy: 25%; cycle time: 0.6 s.

DIA mass spectrometry data were processed using DIA‐NN software, which combined all mass spectrometry data, performed database searches, and conducted protein quantification analyses. Protein expression trends across multiple groups were analyzed using one‐way ANOVA, with significant proteins (*p* < 0.05) visualized.

### 4.19 Sanger Sequencing

The PCR reaction mix included 10–50 ng of genomic DNA, 0.2 µM of each primer, 200 µM of dNTPs, 1.5 mM MgCl_2_, 1× PCR buffer, and 1 unit of Taq DNA polymerase (Thermo Fisher Scientific) in a total volume of 50 µL. The PCR conditions were as follows: Initial denaturation at 95°C for 2 min; 35 cycles of denaturation at 95°C for 30 s, annealing at 55°C for 30 s, and extension at 72°C for 1 min; followed by a final extension at 72°C for 10 min. NanoDrop spectrophotometer (Thermo Fisher Scientific) was used to ensure sufficient concentration for sequencing.

The sequencing reactions were set up in a total volume of 10 µL, containing 1–2 µL of purified PCR product, 1 µL of BigDye Terminator v3.1 Ready Reaction Mix, 1.5 µL of 5 × sequencing buffer, 1 µL of 3.2 pmol µL^−1^ primer, and nuclease‐free water to bring the volume to 10 µL. The cycle sequencing conditions were: Initial denaturation at 96°C for 1 min; followed by 25 cycles of 96°C for 10 s, 50°C for 5 s, and 60°C for 4 min; and a final hold at 4°C.

Post‐sequencing reactions were purified using the ethanol/EDTA precipitation method. Briefly, 1 µL of 125 mM EDTA and 1 µL of 3 M sodium acetate (pH 4.6) were added to the sequencing reaction, followed by 30 µL of 100% ethanol. The mixture was incubated at room temperature for 15 min and then centrifuged at 12,000 rpm for 15 min at 4°C. Then, the supernatant was carefully discarded, and the pellet was washed with 70 µL of 70% ethanol. After a final centrifugation at 12,000 rpm for 5 min, the supernatant was removed, and the pellet was air‐dried. The dried pellet was resuspended in 10 µL of Hi‐Di formamide (Applied Biosystems).

The resuspended samples were subsequently loaded into an Applied Biosystems 3500 Genetic Analyzer. The sequencing runs were performed according to the manufacturer's instructions. The final sequencing data were then visualized by the SnapGene 3.2.1 software to identify the mutations.

### 4.20 Statistics

All the experiments were independently repeated at least twice, and no inconsistent results were observed. Pearson's correlation coefficients (R) were computed using RNA‐seq, CLIP‐seq, and IP‐MS data. Differentially expressed genes in RNA‐seq are identified using the Benjamini–Hochberg test. rMATS turbo software (v4.1.2) is performed to analyze alternative splicing events. To compare the distributions of CLIP‐seq signals between two gene sets, the Kolmogorov–Smirnov test was applied. GraphPad Prism software (version 9.0.0) was used for the statistical analysis. Data are expressed as mean ± SEM. Significant differences between two groups were analyzed using Student's *t*‐test. Statistical significance is shown as follows: exact *p* value *p * ≥  0.05; *p  <  0.05; **p  <  0.01; ***p  <  0.001; ****p  <  0.0001. All the data were reproducible, and details of replicates are described in the figure legends.

## Author Contributions

Yu Chen and Siyuan Sun wrote the original manuscript. Yu Chen and Siyuan Sun generated knocking cell lines and performed genotyping, CLIP‐seq and RNA‐seq, FACS, and immunofluorescence. Chenxu Lu, Yixuan Li, and Bing Fang performed ELISA, qRT‐PCR, and Western blot analysis with the help of Xuepeng Li, Yumei Lei, Longjie Sun, and Ming Zhang. Xiangfeng Tang collected patient samples and clinical data. Weiru Yu and Ping Liu performed GO analysis. Changchang Cao performed the bioinformatic analysis. Jiazeng Sun and Yongting Luo prepared the figures with the help of Xingwang Zhao, Jing Zhan, and Libing Liu. Rong Liu and Jiaqiang Huang advised on bioinformatics analysis. Ziwei Yi, Yifei Yu, Weihan Xiao, Zheng Ding, Lei Li, and Dan Su supervised the experiments. Juan Chen with the help of Wenbiao Shi, Ran Wang, and Fazheng Ren initiated the study and planned the study. Juan Chen, Wenbiao Shi, Ran Wang, and Fazheng Ren wrote the final manuscript.

## Conflicts of Interest

The authors declare no conflicts of interest.

## Supporting information



Supporting Information

Supporting Information

Supporting Information

Supporting Information

Supporting Information

## Data Availability

RNA‐seq and CLIP‐seq data have been deposited at Gene Expression Omnibus under the accession number GSE271398.
