# Supplementary material for: The RNA Binding Protein Bcas2 is Required for Antibody Class Switch in Activated‐B Cells
Source: Exploration (Beijing). 2025 Feb 16;5(3):270015. doi: 10.1002/EXP.70015 (PMC12199426; doi:10.1002/EXP.70015)
Supplement: Supplementary file 1 — Supporting Information [file EXP2-5-270015-s001.docx]

**Supplementary Materials**

**Supplementary Table 1**. List of identified proteins by serum proteomics.

**Supplementary Table 2**. Primers used in this study.

**Supplementary Table 3**. List of Target Genes Identified by CLIP-Seq.

**Supplementary Table 4**. List of Differentially Expressed Genes in RNA-Seq between WT and *Bcas2*-cKO.


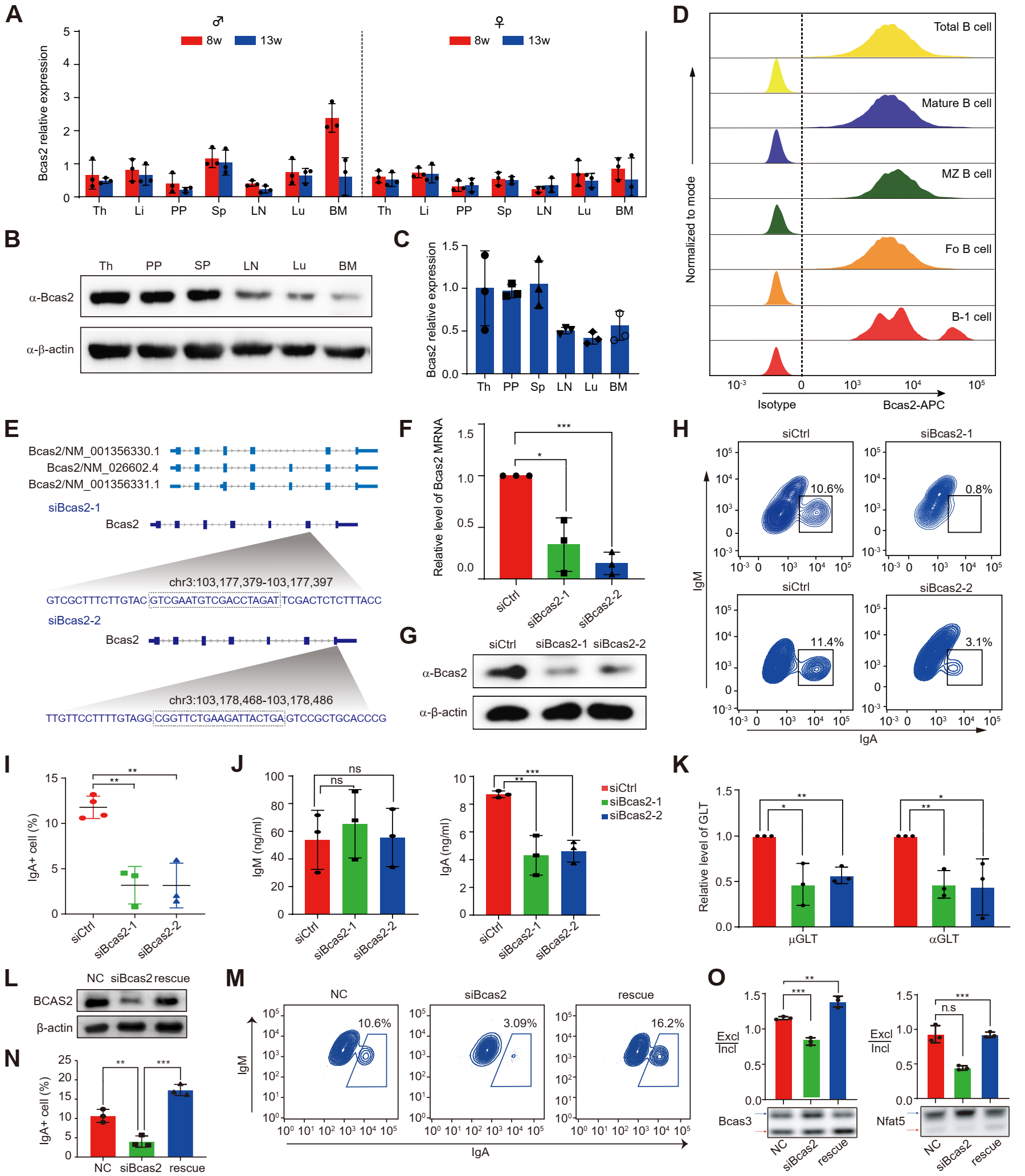
**Figure S1 Bcas2 expression in different types of B cells in the spleen. A,** Quantitative PCR analysis of mRNA from different organs of 8-week-old and 13-week-old female and male WT mice. Bcas2 expression levels were normalized to β-actin transcripts in quantitative PCR analysis (n = 3, mean ± SD). **B,** Western blot in different organs of 8-week-old male mice. **C,** Relative expression of Bcas2 from different organs of male mice at 8 weeks old WT mice. Bcas2 expression levels were normalized to β-actin (n = 3, mean ± SD). **D,** Flow cytometric analysis of the relative expression of Bcas2 within different types of B cells in the spleen. **E,** A schematic diagram illustrating the transcript variants of Bcas2 and the locations of two Bcas2 siRNA sequences. **F,** Quantitative PCR analysis of B cell mRNA of siCtrl and two Bcas2 siRNA groups. Bcas2 expression levels were normalized to Gapdh transcripts and the siCtrl group in quantitative PCR analysis (n = 3, mean ± SD). **G,** Western blot of B cell mRNA of siCtrl and two Bcas2 siRNA groups. **H,** Flow cytometric analysis for CSR in CH12 cell lines of NC and two Bcas2 siRNA groups after CIT stimulation. **I,** Quantification of CSR shown in (H). Each symbol represents CH12 cell cultures (n ≥ 3, mean ± SD). **J,** Concentrations of IgM and IgA in CH12 cell culture supernatant from siCtrl and two Bcas2 siRNA groups. Each symbol represents technical duplications and columns indicate the mean. **K,** Quantitative PCR analysis of mRNA after 24 hr in CIT stimulation. Expression levels of μGLT and aGLT were normalized to β-actin transcripts and the siCtrl group of CIT stimulation (n = 3, mean ± SD). **L,** Bcas2 protein expression in Bcas2 knockdown and rescue groups. **M,** Flow cytometric analysis for CSR in CH12 cell lines with Bcas2 knockdown and rescue. **N,** Quantification of CSR shown in (M). Each symbol represents CH12 cell cultures (n = 3, mean ± SD). **O,** The alternative splicing events in CH12 cell lines with Bcas2 knockdown and rescue.


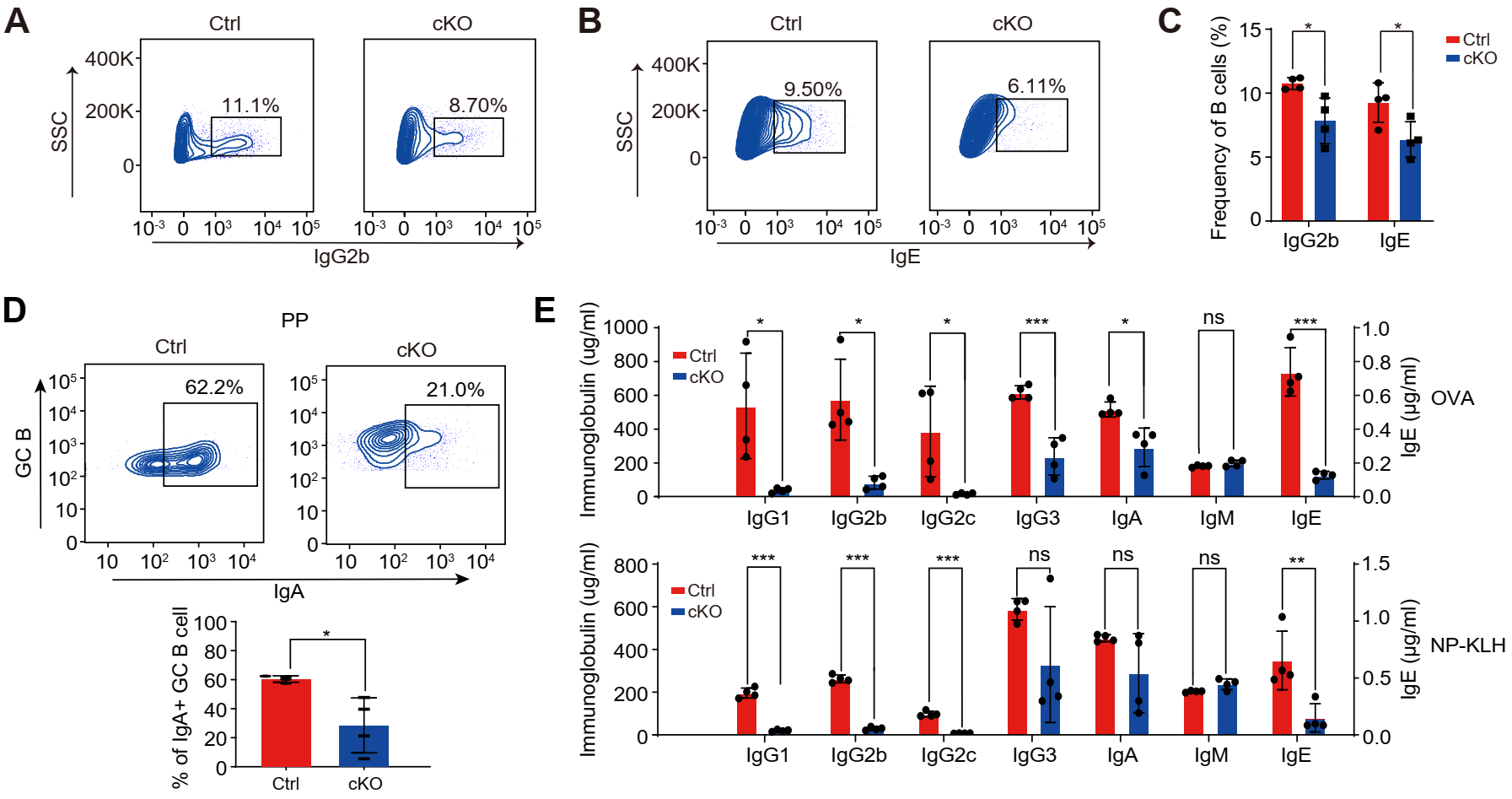
**Figure S2 Bcas2 influences class switch recombination (CSR) in mice following immunization with OVA and NP-KLH. A,** Flow cytometric analysis for surface IgG2b expression after LPS stimulation. **B,** Flow cytometric analysis for surface IgE expression after LPS+IL4 stimulation. **C,** Quantification of IgG2b and IgE for LPS and LPS+IL4 cultures shown in (A) and (B) (n > 4, mean ± SD). **D,** Flow cytometric analysis of the proportions and surface IgA expression of GC B cell populations in the Peyer's patches of WT and *Bcas2*-cKO mice. Each symbol represents B cell cultures from individual mice and columns indicate the mean (n ≥ 3, mean ± SD). **E,** Concentrations of IgG1, IgG2b, IgG2c, IgG3, IgA, IgM, and IgE in WT and *Bcas2*-cKO mice serum after OVA or NP-KLH immunization.


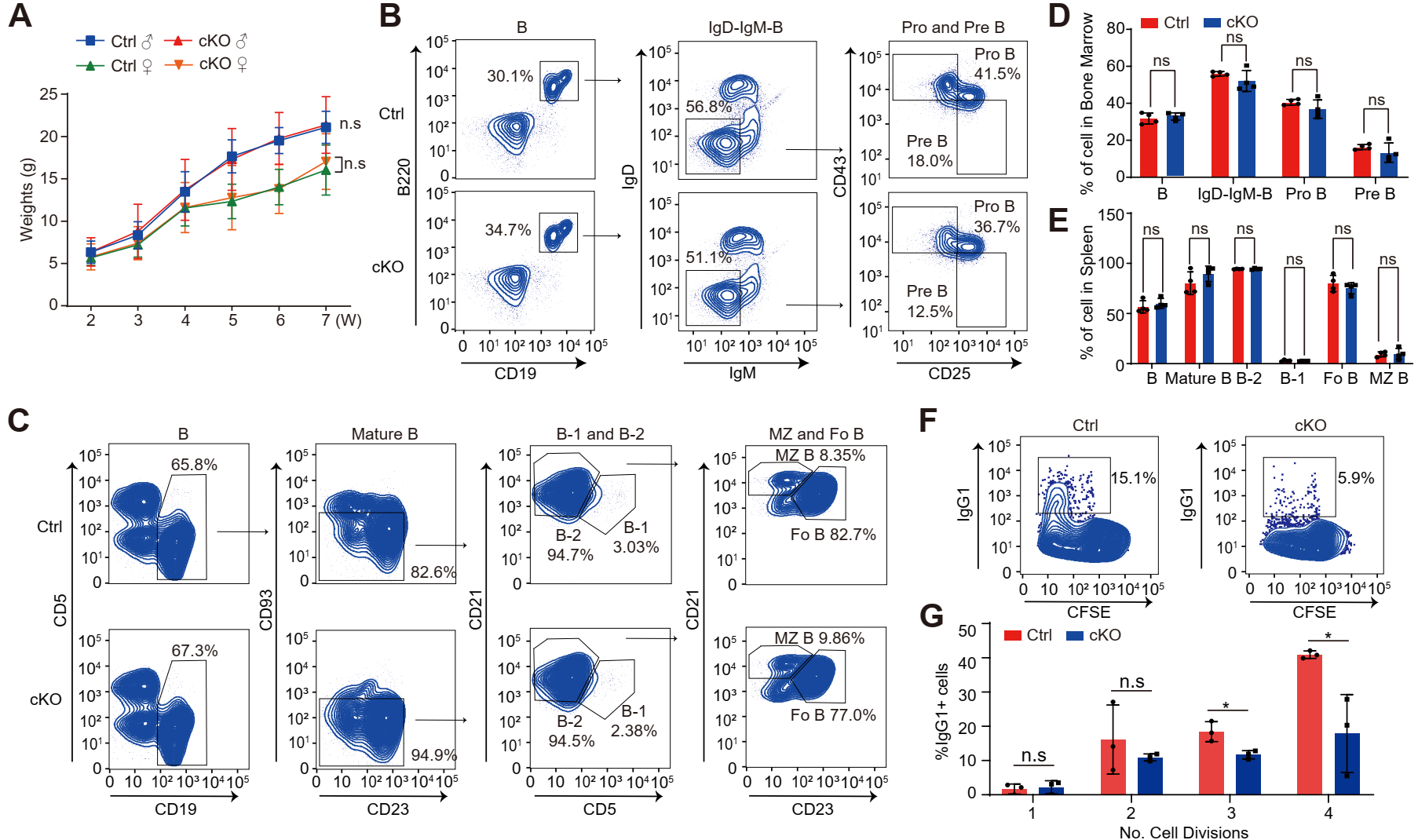
**Figure S3 AID-mediated specific knockout of Bcas2 does not affect B cell development. A,** Line graph illustrating the weight changes of female and male CTRL and *Bcas2*-cKO mice. **B,** Flow cytometric analysis of the proportions of various B cell populations in the spleens of WT and *Bcas2*-cKO mice. **C,** Flow cytometric analysis of the proportions of various B cell populations in the bone marrow of WT and *Bcas2*-cKO mice. **D,** Quantification of various B cells shown in (B). Each symbol represents B cell cultures from individual mice (n = 4, mean ± SD). **E,** Quantification of various B cell shown in (C). Each symbol represents B cell cultures from individual mice (n = 4, mean ± SD). **F**, Flow cytometric analysis for surface IgG1 expression and CFSE in WT and Bcas2 cKO B cells stimulated for 3 days. **G**, Percentage of IgG1^+^ cells analyzed per number of cell divisions in CFSE-labeled LPS plus IL-4 cultures at day 3. One representative experiment with 3 mice per genotype (mean ± SD)


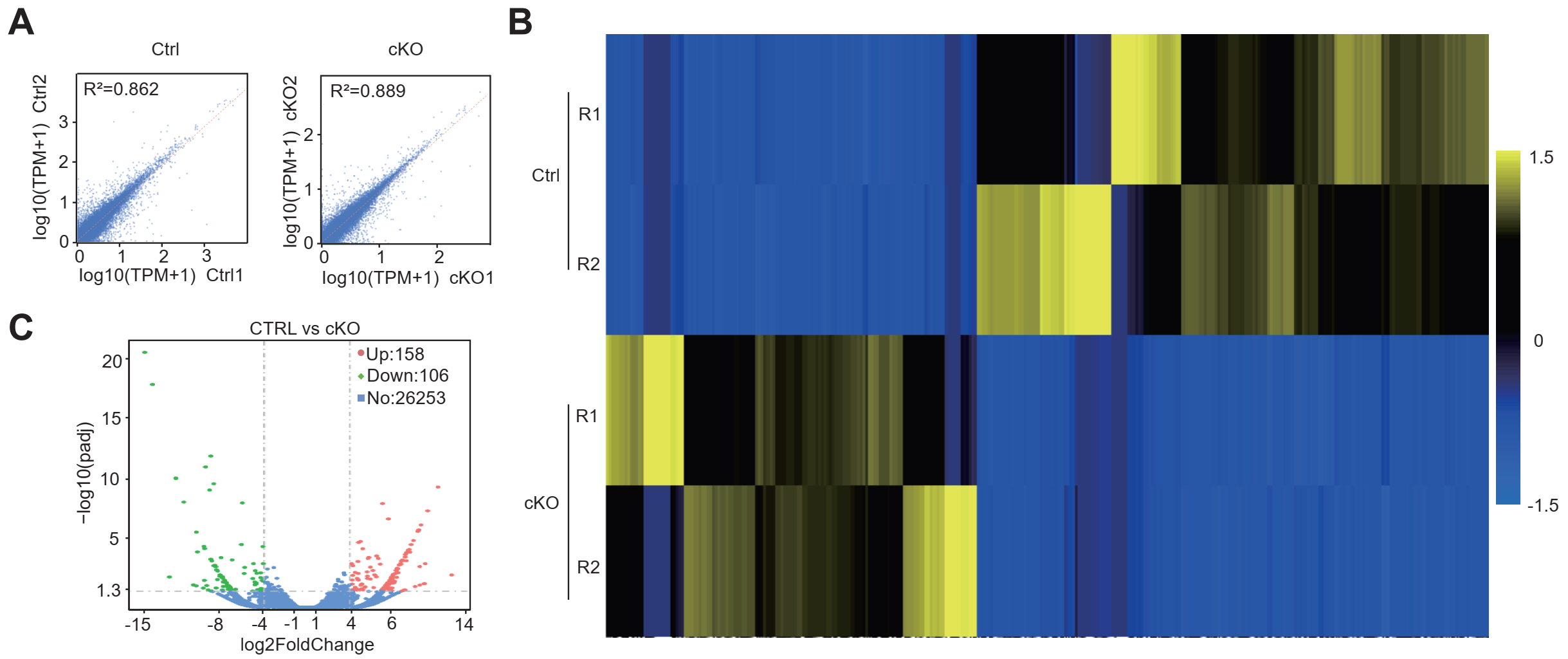


**Figure S4 Bcas2 regulates lncRNA abundance. A,** Pearson correlation analysis shows the coefficients between two replicates of WT and *Bcas2*-cKO mice in the lnc RNA-seq data. **B,** Volcano map displaying the distribution of differentially expressed genes from lnc RNA-seq data. The abscissa in the figure represents the gene fold change in LPS+IL4 stimulated WT and *Bcas2*-cKO B cells. |FoldChange| ≥ 1.5. Padj ≤ 0.05. The ordinate indicates the significance of gene expression differences between LPS+IL4 stimulated WT and *Bcas2*-cKO B cells. Upregulated genes are shown as red dots, and downregulated genes are shown as green dots. **C,** Cluster heatmap of differentially expressed genes. The abscissa is the genotype, and the ordinate is the normalized FPKM (fragments per kilobase million) value of the differentially expressed gene. Yellow indicates a higher expression level, while blue indicates a lower expression level.


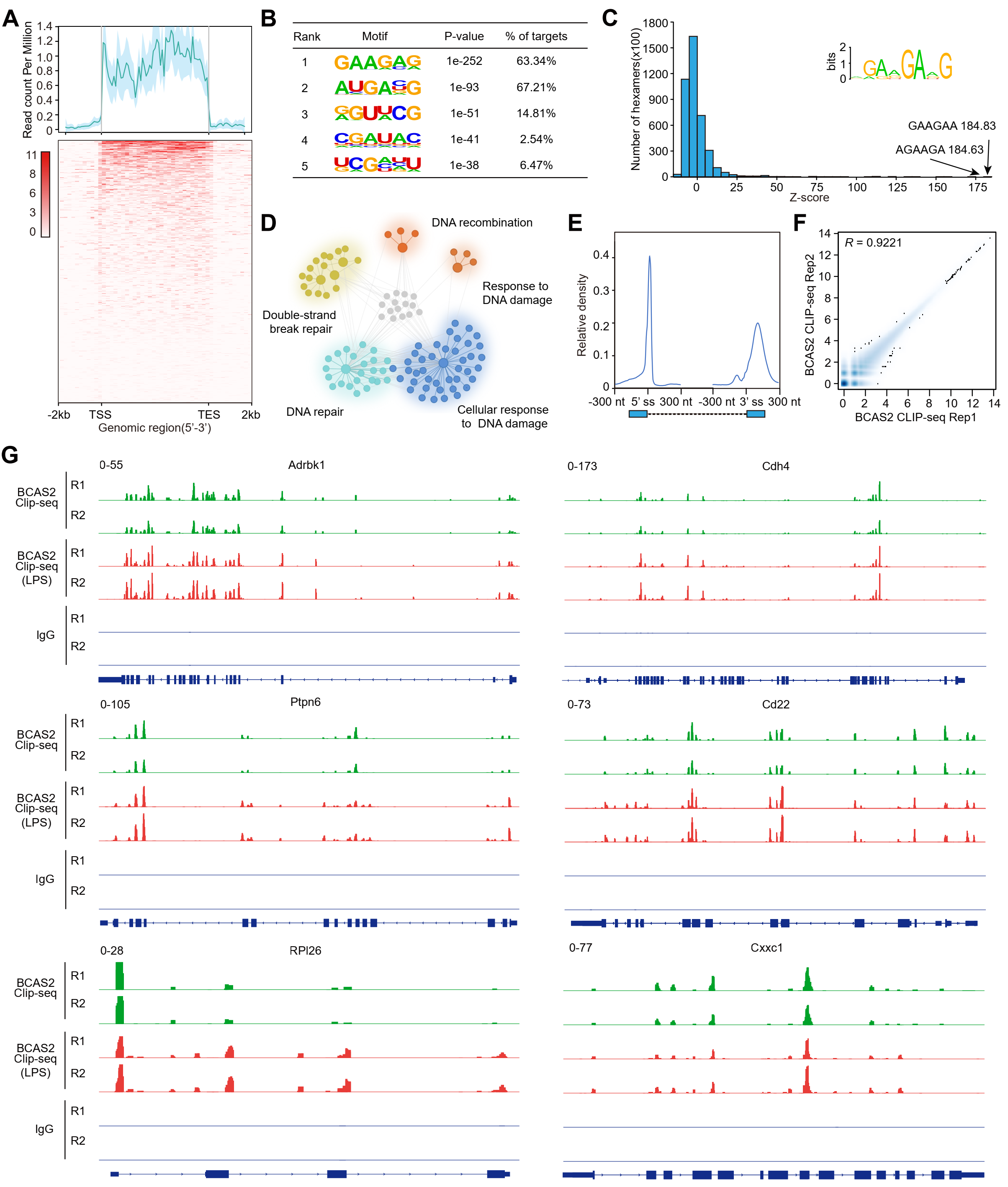
**Figure S5 Bcas2 directly binds mRNA in spleen. A,** Meta-analyses showed the binding of Bcas2 in the genomic region between TSS and TES. **B,** Enriched Bcas2 binding motifs. The top five enriched motifs are shown. **C,** Histogram showing overrepresented Bcas2-binding motifs identified by CLIP-seq. The Z scores of the top two hexamers are indicated. The insert shows the Bcas2-binding consensus calculated from the top 20 enriched hexamers. **D,** GO enrichment analysis network showing the corresponding genes for Bcas2 binding RNA. **E,** Meta-analysis showing the relative density of Bcas2 binding to 5'ss and 3'ss. **F,** Pearson's correlation analysis shows the coefficient between two replicates in the Clip-seq data. **G,** The Bcas2-binding peaks of CSR-related gene transcripts are shown. Purple arrow, direction of transcription. F, forward primers. R, reverse primers.


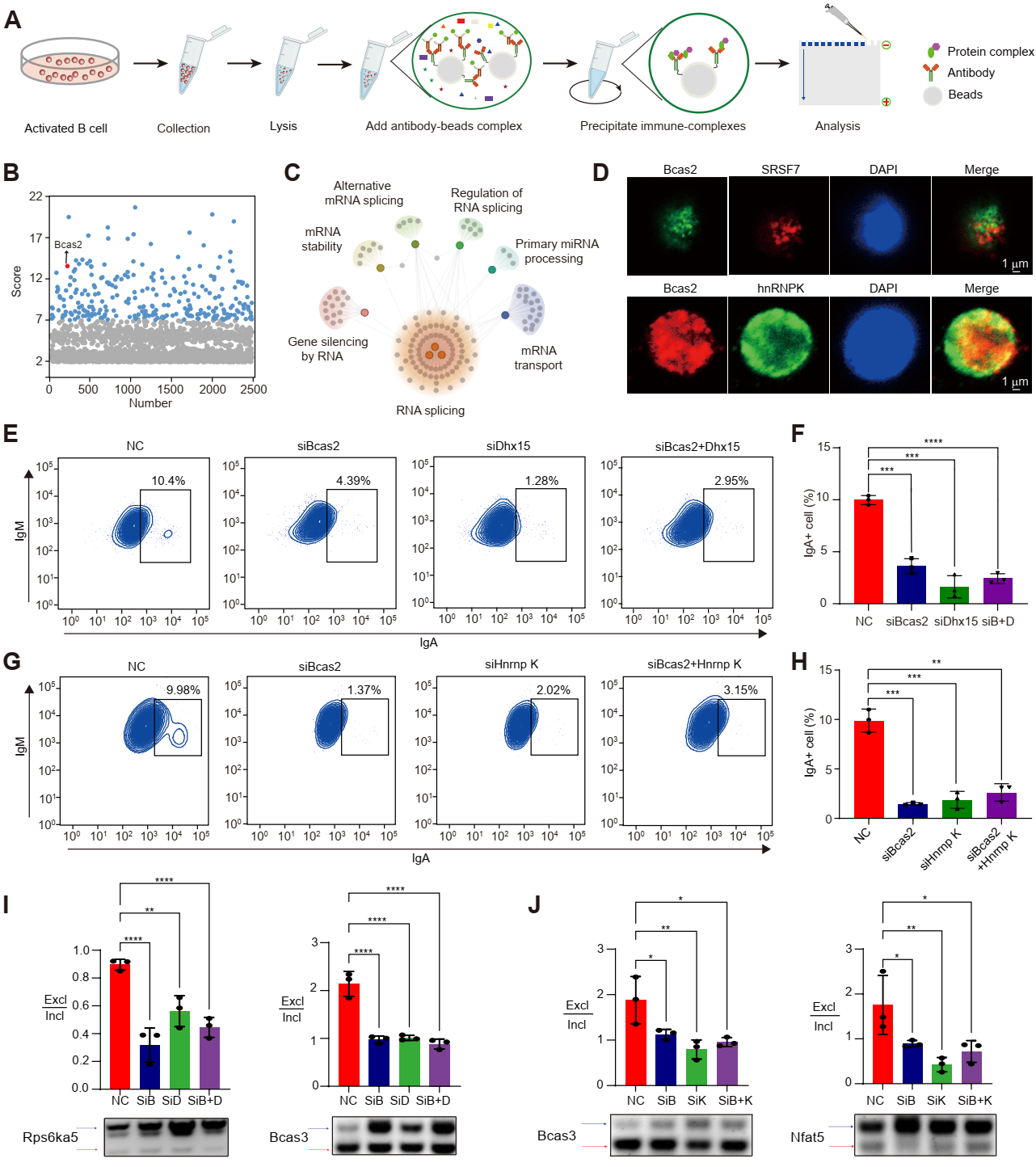
**Figure S6 Bcas2 exhibits similar protein recruitment in primary B cells. A,** Schematic diagram showing the flow of the co-immunoprecipitation. **B,** Scatter plot of protein scores demonstrate significant differences in proteins between two groups of parallel mixed samples. **C,** Network showing GO enrichment analyses of Bcas2-binding proteins. **D,** Co-immunostaining was performed using Bcas2 and Bcas2-binding proteins (SRSF7 and hnRNP K) antibodies from splenic B cells of adult mice. DNA was stained with DAPI. Scale bar, 1μm. **(E, G)**, Flow cytometric analysis for CSR in CH12 cell lines with Bcas2 and Dhx15 knockdown or Bcas2 and Hnrnp K knockdown after CIT stimulation. **(F, H)** Quantification of CSR shown in (E, G). Each symbol represents CH12 cell cultures (n = 3, mean ± SD). **(I, J)** the alternative splicing events of CH12 cells with Bcas2 and Dhx15 knockdown and of Bcas2 and Hnrnp K knockdown.


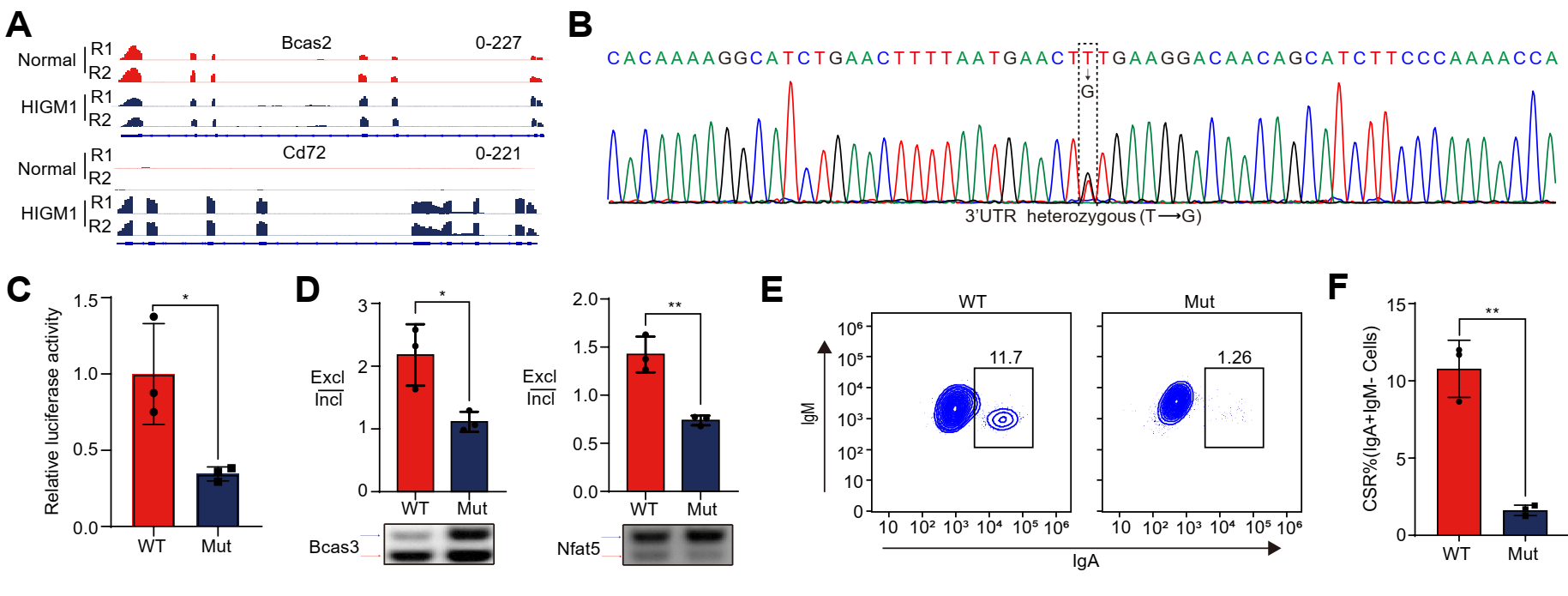


**Figure S7 3’UTR mutation of Bcas2 contributes to changes in alternative splicing events and the ability of class-switch recombination. A,** RNA-seq data represent the expression of Bcas2 and Cd72 between normal people and HIGM1 patients. **B,** Sanger sequencing results of the target gene region. Each peak represents a nucleotide, and the height of the peak reflects the relative abundance of the nucleotide. **(C, D)** mRNA expression in wild type cells and cells with Bcas2 mutant plasmid, and the alternative splicing events of Bcas3 and Nfat5, respectively. **E,** Flow cytometric analysis for CSR in CH12 cell lines after CIT stimulation. **F,** Quantification of CSR shown in (E). Each symbol represents CH12 cell cultures (n ≥ 3, mean ± SD).


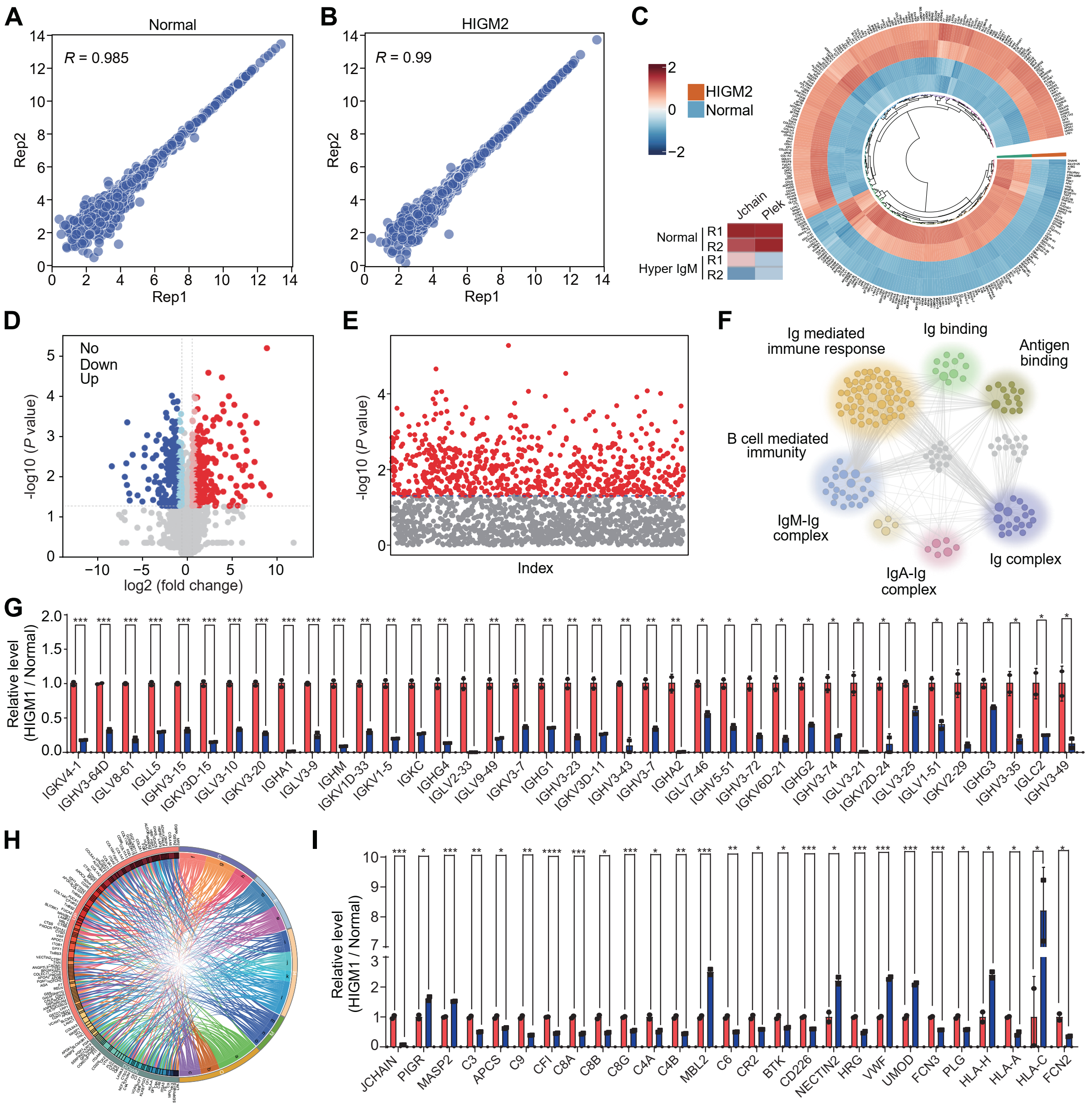
**Figure S8 Reduced expression of heavy chain antibodies in the serum of Hyper IgM patients. (A-B),** Pearson’s correlation analysis shows the coefficient between two replicates in the sample of the normal people (A) and the patient (B) in serum proteomics. **C,** Circos representing the correlation between different proteins and normal people or the HIGM1 patient; with a Cluster heatmap of several proteins related to antibody formation. Red indicates a higher expression level, while blue indicates a lower expression level. **D,** Volcano map displaying the distribution of differentially expressed proteins from proteomics data. The abscissa in the figure represents the protein fold change in serum of normal human and Hyper IgM patient. |FoldChange|≥ 1.5. Padj ≤ 0.05. The ordinate indicates the significance of gene expression differences between the HIGM patient and normal subjects. Upregulated genes are shown as red dots, and downregulated genes are shown as blue dots. **E,** Scatter plot of differentially expressed proteins. The abscissa is the protein indexes, and the ordinate is the -log10 (P value) of the differentially expressed protein. Red indicates a higher expression level above the threshold with the -log10 (P value) = 1.5. **F,** Network showing GO enrichment analyses of differentially expressed proteins of Hyper IgM patient serum. **G,** Relative levels of different Ig heavy chains in the serum of normal individuals compared to Hyper IgM patients. All the relative expression levels of peptides in normal people were normalized to 1. **H,** Protein regulation network in serum proteomics. **I,** Differentially expressed proteins of Hyper IgM patient from serum proteomics. All the relative expression levels of proteins in normal people were normalized to 1.
